# Enhanced Photocatalytic Performance for CO_2_ Reduction Using an Indirect Z‐Scheme Heterojunction Photocatalyst

**DOI:** 10.1002/cssc.70716

**Published:** 2026-05-12

**Authors:** I‐Hua Tsai, Chen‐Hsiu Fu, Ting‐Hui Lin, Shu‐Yu Lin, Eric Wei‐Guang Diau

**Affiliations:** ^1^ Department of Applied Chemistry Institute of Molecular Science National Yang Ming Chiao Tung University Hsinchu Taiwan; ^2^ Center for Emergent Functional Matter Science National Yang Ming Chiao Tung University Hsinchu Taiwan

**Keywords:** Ag nanoparticles, bismuth oxyiodide (BiOI), graphitic carbon nitride (g‐C3N4), indirect Z‐scheme heterojunction, photocatalytic CO_2_ reduction

## Abstract

Efficient suppression of charge recombination remains a central challenge in photocatalytic CO_2_ reduction. Here, we report a rational source‐to‐design strategy to construct both direct and indirect Z‐scheme heterojunctions by integrating graphitic carbon nitride (*g*‐C_3_N_4_), bismuth oxyiodide (BiOI), and Ag nanoparticles. A solvent‐free ball‐milling process combined with light‐driven Ag photodeposition enables intimate interfacial coupling while preserving the layered frameworks of both semiconductors. Among the resulting systems, the Ag‐bridged indirect Z‐scheme exhibits a CO evolution yield of 344.6 μmol g^−1^ under visible‐light irradiation, markedly outperforming pristine *g*‐C_3_N_4_ and the direct Z‐scheme counterpart. Mechanistic investigations reveal that Ag nanoparticles function as efficient electron mediators, facilitating directional electron transfer from BiOI to Ag and hole transfer from *g*‐C_3_N_4_ to Ag, thereby suppressing recombination and enhancing carrier mobility. This work establishes an effective design paradigm for indirect Z‐scheme photocatalysts and provides general insights into mediator‐assisted interfacial engineering for solar‐driven CO_2_ conversion.

## Graphical abstract

A photocatalyst system has been constructed for CO_2_ reduction reaction using graphitic carbon nitride (*g*‐C_3_N_4_) as reduction center, bismuth oxyiodide (BiOI) as oxidation center via hole scavenger TEOA, and Ag nanoparticle as hole–electron recombination mediate. This indirect Z‐scheme system has the outstanding photocatalytic activity than the conventional Z‐scheme system.

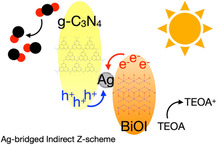

## 1 Introduction

Since the industrial revolution, the intensive combustion of fossil fuels has accelerated technological and economic growth while simultaneously driving severe greenhouse gas emissions and global climate change. Carbon dioxide (CO_2_), the most abundant anthropogenic greenhouse gas, has increased from ≈280 ppm in the preindustrial era to over 420 ppm today, contributing to extreme weather events, ecological disruption, and socioeconomic risks [1, 2]. To address these challenges, numerous strategies, including carbon capture [3], storage [4], and catalytic conversion, have been developed. Among them, photocatalytic CO_2_ reduction reaction (CO_2_RR) is particularly appealing due to its dual advantages of achieving carbon neutrality while converting CO_2_ into value‐added chemical products [5, 6].

Photocatalysis operates under mild reaction conditions using sunlight as the sole energy input, distinguishing it fundamentally from thermocatalytic and electrocatalytic approaches [7, 8, 9]. Nevertheless, its practical efficiency is often limited by three intrinsic factors: insufficient visible‐light absorption, rapid recombination of photogenerated charge carriers, and sluggish surface reaction kinetics [10, 11, 12]. Consequently, the rational design of semiconductor photocatalysts with improved light harvesting, charge separation, and interfacial reaction efficiency remains a central objective in artificial photosynthesis research.

Graphitic carbon nitride (*g*‐C_3_N_4_) has emerged as a representative metal‐free photocatalyst owing to its moderate bandgap (≈2.7 eV), high thermal and chemical stability, and scalable synthesis from earth‐abundant precursors [13, 14, 15]. Despite these advantages, pristine *g*‐C_3_N_4_ suffers from limited visible‐light absorption beyond ≈460 nm and severe charge recombination associated with its layered structure and intrinsic defects, resulting in modest CO_2_RR performance. To overcome these limitations, extensive efforts have focused on defect engineering, elemental doping, and heterojunction construction. Among various coupling partners, bismuth oxyiodide (BiOI), a p‐type layered semiconductor with a narrow bandgap (1.8–2.1 eV) and strong internal electric field, has attracted considerable interest[13, 16, 17]. Previous studies have demonstrated that BiOI/*g*‐C_3_N_4_ heterojunctions can enhance photocatalytic activity through favorable band alignment and interfacial charge separation [18, 19]. However, further improvement is often hindered by limited carrier lifetime and insufficient reduction potential.

To overcome these drawbacks, Z‐scheme heterojunctions have been proposed as an advanced photocatalytic architecture capable of simultaneously suppressing charge recombination and preserving the strong redox potentials of individual semiconductors [20, 21, 22, 23, 24, 25]. Inspired by natural photosynthesis, Z‐scheme systems enable selective recombination of low‐energy charge carriers, thereby retaining highly reducing electrons and strongly oxidizing holes for redox reactions [26]. Z‐scheme heterojunctions can be broadly classified as direct and indirect configurations. In direct Z‐scheme systems, interfacial recombination is driven by band bending and internal electric fields at the semiconductor interface. In contrast, indirect Z‐scheme systems employ conductive mediators, such as metals or carbon‐based materials, to facilitate directional charge transfer and spatially separate photogenerated carriers [27, 28, 29, 30, 31].

Recent studies have demonstrated that *g*‐C_3_N_4_‐based Z‐scheme and S‐scheme heterojunctions can significantly enhance CO_2_ reduction performance through interfacial electronic reconstruction [32, 33]. However, most reported systems focus on a single configuration, and systematic comparisons between direct and indirect Z‐scheme architectures constructed from identical components under identical conditions remain limited.

The BiOI/*g*‐C_3_N_4_ system provides an ideal model platform for such a comparison, as distinct charge transfer architectures can be realized through controlled synthetic routes. Although BiOI/*g*‐C_3_N_4_ heterostructures incorporating Ag or AgI have been reported [34], these systems often involve AgI as an additional photoactive phase or emphasize plasmonic enhancement, complicating isolation of the architectural effect. In contrast, the present work focuses on an architecture‐controlled comparison between direct and metal‐bridged indirect Z‐scheme BiOI/*g*‐C_3_N_4_ heterojunctions constructed from the same semiconductor pair. A solvent‐free mechanochemical ball‐milling strategy is employed to establish intimate interfacial contact without solution‐mediated ion exchange, followed by light‐driven deposition of metallic Ag to form a conductive interfacial bridge. Under identical photocatalytic conditions, the charge transfer behaviors and CO_2_ reduction performances of these architectures are systematically evaluated. This study provides mechanistic insight into mediator‐assisted indirect Z‐scheme photocatalysis and demonstrates interfacial energy‐band engineering as a rational design strategy for next‐generation *g*‐C_3_N_4_‐based photocatalysts.

## 2 Results and Discussion

**FIGURE 1:**
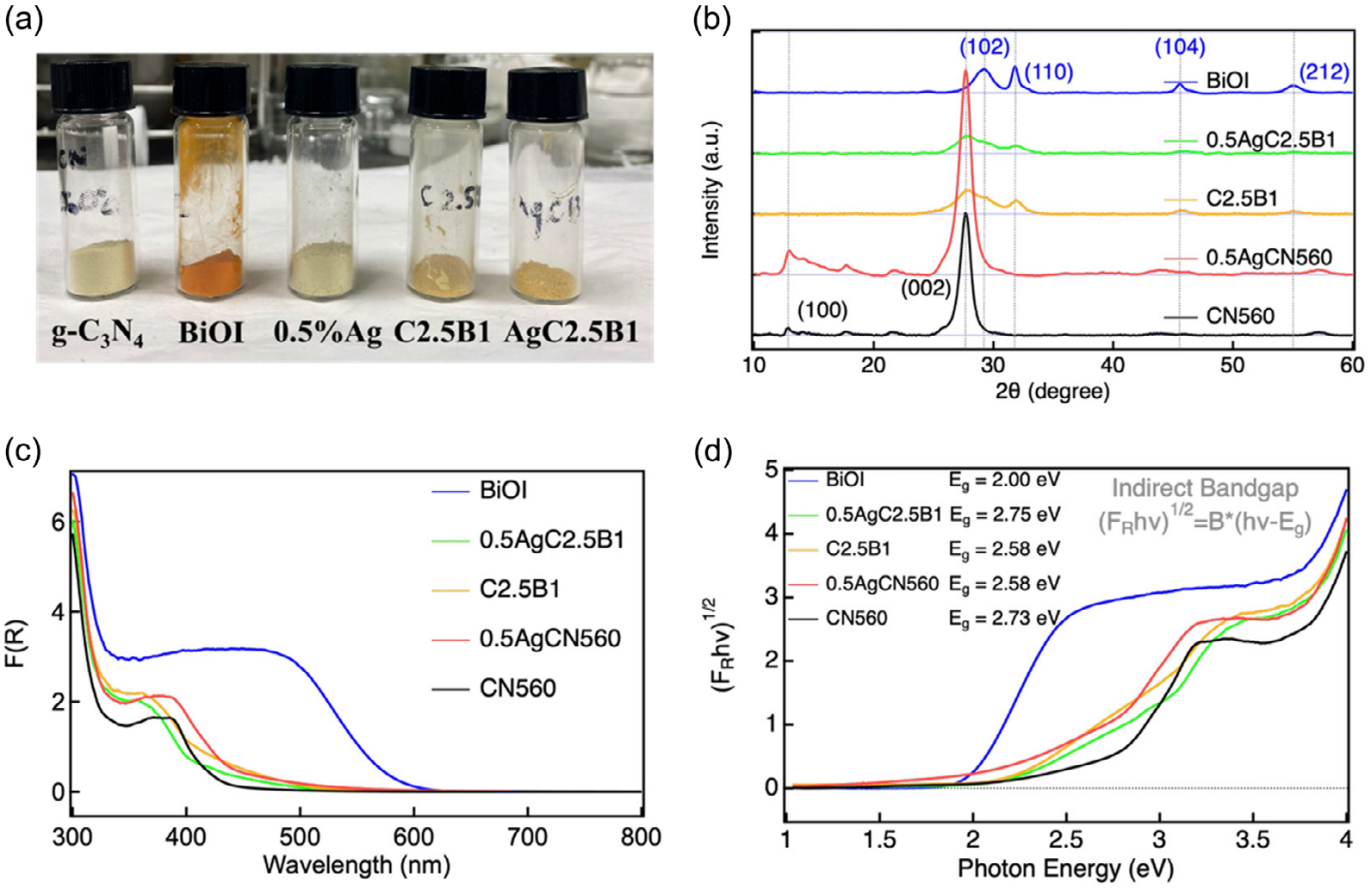
(a) Photographs of pristine *g*‐C_3_N_4_ (CN560), BiOI, Ag‐deposited *g*‐C_3_N_4_ (0.5AgCN560), direct Z‐scheme composite (C2.5B1), and indirect Z‐scheme composite (0.5AgC2.5B1) powders under indoor illumination. (b) XRD patterns of the five samples. (c) UV–Vis diffuse reflectance spectra after Kubelka–Munk correction. (d) Tauc plots used to estimate optical bandgaps. XRD = X‐ray diffraction.

The experimental details are provided in the Supporting Information (SI). The discussion below focuses on samples prepared under optimized conditions. CN560 denotes *g*‐C_3_N_4_ synthesized at 560°C; 0.5AgCN560 refers to CN560 modified with 0.5 wt% Ag via photodeposition; C2.5B1 represents a direct BiOI/*g*‐C_3_N_4_ composite prepared at a weight ratio of 2.5:1; and 0.5AgC2.5B1 corresponds to the Ag‐bridged indirect Z‐scheme composite derived from C2.5B1. Figure 1a displays photographs of CN560, BiOI, 0.5AgCN560, C2.5B1, and 0.5AgC2.5B1 powders under ambient light. The gradual color evolution from pale yellow (CN560) to reddish‐brown (BiOI), with intermediate tones for the composites, qualitatively reflects progressive modulation of light absorption upon heterojunction formation.

The optimization of *g*‐C_3_N_4_ synthesis temperature is shown in Figures S1–S4 and Tables S1–S2 [35]. X‐ray diffraction (XRD) analysis reveals that increasing the calcination temperature from 540 to 580°C induces a systematic shift of the (002) reflection from 27.5° to 27.7°, accompanied by a decrease in the interlayer spacing from 3.25 to 3.22 Å (Figure S3), indicating tighter stacking along the *c*‐axis. Concurrently, the full width at half maximum of the (002) peak reaches a minimum at 560°C, evidencing improved crystallinity and reduced microstrain, as quantified by Voigt fitting (Table S1). The (100) reflection exhibits a slight red shift (Figure S2), suggesting partial in‐plane rearrangement of the heptazine units. Photocatalytic CO_2_ reduction tests (Figure S4; Table S2) further show that although CN540 delivers higher initial CO yields, its reproducibility is poor, whereas CN560 provides the most consistent performance with minimal deviation. Accordingly, CN560 was selected as a reliable structural and catalytic baseline for subsequent composite construction.

The XRD patterns of the composite systems are shown in Figure 1b. Pristine BiOI retains its tetragonal phase [36], with characteristic reflections indexed to the (102), (110), (104), and (212) planes, while the CN560‐derived reflections remain in all composites. Notably, the in‐plane (100) reflection of *g*‐C_3_N_4_ at ≈13° becomes markedly weakened or even undetectable in the C2.5B1‐related samples, accompanied by reduced intensity of the (002) peak. This behavior indicates a loss of long‐range in‐plane ordering of *g*‐C_3_N_4_ induced by mechanochemical treatment and intimate interfacial contact with BiOI, rather than complete degradation of the graphitic framework. The persistence of the (002) reflection confirms that the layered stacking motif of *g*‐C_3_N_4_ is largely preserved, while its lateral periodicity is partially disrupted by interfacial reconstruction and surface coverage. No additional diffraction peaks are observed after composite formation or Ag photodeposition, and no reflections attributable to AgI or other Ag halides are detected within the instrumental resolution, indicating that Ag remains predominantly metallic. The slightly increased intensity of the *g*‐C_3_N_4_ (002) reflection in 0.5AgCN560 is attributed to variations in sample packing, preferred orientation, and stacking coherence during photodeposition and drying. Although diffraction intensity can be influenced by crystallinity, the observed change is more reasonably ascribed to these geometric factors rather than to a substantial improvement in intrinsic crystal quality. In contrast, the BiOI‐containing composites undergo stronger interfacial reconstruction and orientation randomization, so no similar enhancement of the (002) reflection is observed. A slight shift of BiOI reflections toward higher 2*θ* values is observed for the ball‐milled C2.5B1 sample, suggesting lattice contraction and denser interfacial packing, a feature that is favorable for interfacial charge transport. Comparative XRD analyses of hydrothermal and ball‐milled composites (Figures S5, S6) further confirm that the crystal frameworks of both CN560 and BiOI remain intact during processing.

The UV–Vis diffuse reflectance spectra (Figure 1c) show systematic evolution of optical absorption across the sample series [37]. Pristine CN560 absorbs predominantly in the UV region with an absorption edge near 400 nm. Ag photodeposition introduces a broad absorption feature in the visible region (Figure S7), consistent with the presence of metallic Ag nanoparticles on the *g*‐C_3_N_4_ surface [38, 39]. With increasing Ag loading, the overall visible‐light absorption is gradually enhanced, leading to an extended absorption tail between 500 and 600 nm. For BiOI‐containing samples (Figures S8–S9), visible‐light absorption in the 450–600 nm range increases with BiOI content, confirming effective optical hybridization of the two semiconductors. Notably, the indirect Z‐scheme composite 0.5AgC2.5B1 exhibits a suppressed baseline in the 600–800 nm region compared to C2.5B1, suggesting reduced diffuse scattering and more compact particle aggregation, consistent with its enhanced photocurrent stability discussed below.

The corresponding Tauc plots (Figure 1d) provide quantitative bandgap estimations [40]. CN560 exhibits an indirect bandgap of 2.73 eV, whereas BiOI shows a narrower gap of 2.00 eV. Upon Ag modification, 0.5AgCN560 displays an apparent red shift to 2.58 eV (Figure S10; Table S3), reflecting electronic coupling between *g*‐C_3_N_4_ and Ag. Both hydrothermal and ball‐milled BiOI/*g*‐C_3_N_4_ composites show comparable bandgap narrowing (Figures S11, S12; Tables S4, S5), indicative of interfacial electronic hybridization. Interestingly, the indirect Z‐scheme 0.5AgC2.5B1 exhibits a slightly wider apparent bandgap, accompanied by reduced Urbach tailing, implying suppression of defect‐related sub‐bandgap states and more ordered electronic delocalization.

Overall, the combined structural and optical analyses demonstrate that Ag deposition and BiOI incorporation systematically modulate lattice order, interfacial contact, and electronic structure without inducing framework collapse. These modifications are therefore discussed primarily as manifestations of controlled electronic coupling and structural compactness, rather than as direct evidence of plasmon‐driven catalytic enhancement. The resulting architecture provides a robust foundation for efficient interfacial charge transfer in subsequent photocatalytic CO_2_ reduction processes.

**FIGURE 2:**
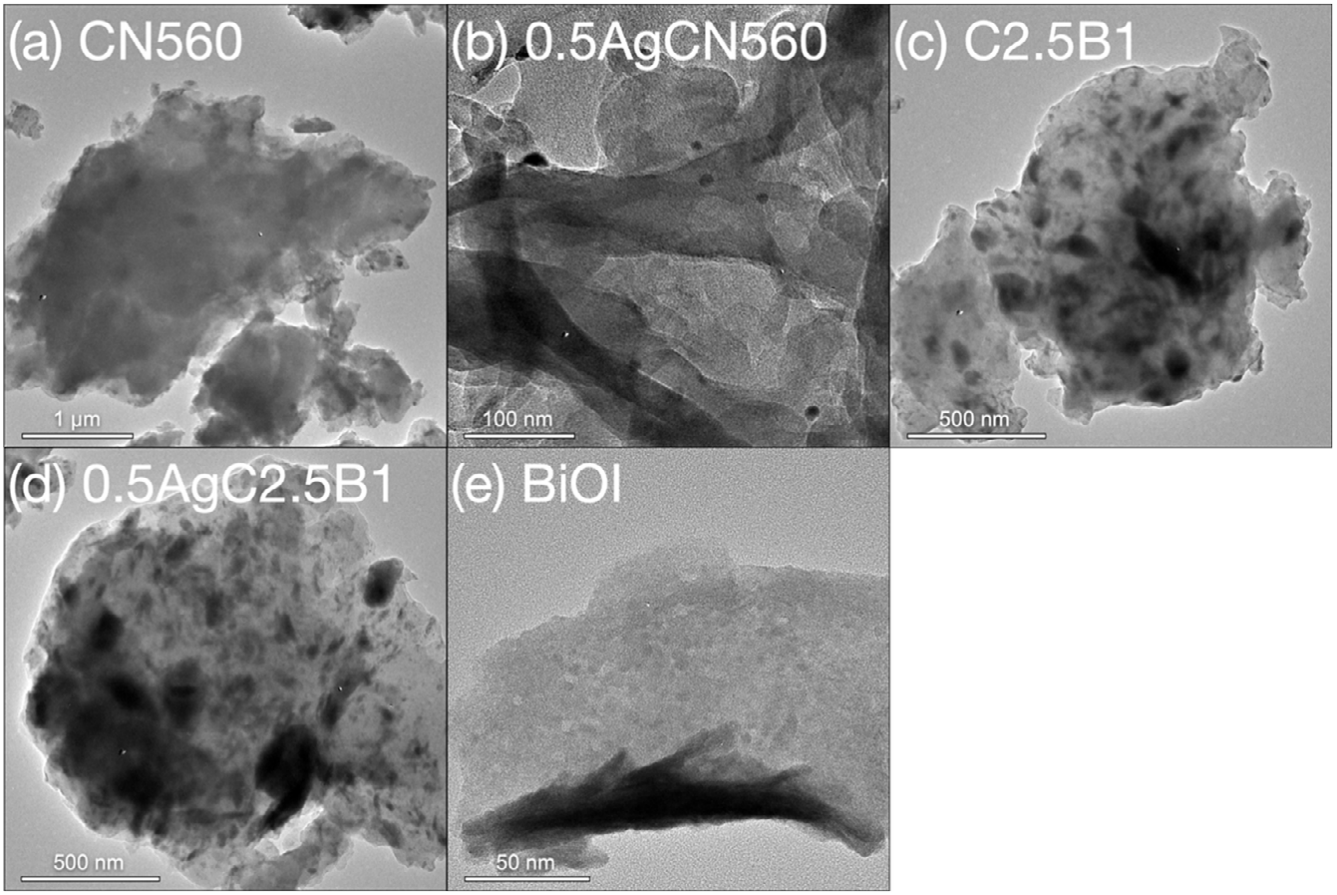
TEM images of (a) CN560, (b) 0.5AgCN560, (c) C2.5B1, (d) 0.5AgC2.5B1, and (e) BiOI. TEM = Transmission electron microscopy.

The transmission electron microscopy (TEM) images in Figure 2 illustrate the morphological evolution from pristine components to the integrated heterojunction architectures. Pristine CN560 consists of thin, stacked nanosheets with a typical layered morphology (Figure 2a), while BiOI exhibits well‐defined lamellar platelets characteristic of its tetragonal layered structure (Figure 2e). After Ag photodeposition, uniformly dispersed spherical Ag nanoparticles are observed on the CN560 surface (Figure 2b), with an average diameter of 7.5 ± 2.3 nm (Figure S13). The absence of large aggregates indicates efficient in situ photoreduction and homogeneous nucleation of metallic Ag on *g*‐C_3_N_4_. In the direct Z‐scheme composite C2.5B1 (Figure 2c), both CN560 and BiOI retain their lamellar morphologies after mechanochemical treatment; however, the two phases exhibit extensive overlap and intimate contact, manifested as darker interfacial contrast at the nanoscale. This observation confirms that ball milling induces close interfacial coupling rather than simple physical mixing. Such mechanically enforced contact is consistent with the slight shift of BiOI diffraction peaks in XRD (Figure 1b), indicative of interfacial lattice compression and strengthened mechanical interlocking. This compact interface is expected to shorten carrier migration distances and enhance interfacial charge transfer probability. The indirect Z‐scheme composite 0.5AgC2.5B1 (Figure 2d) displays a similar lamellar framework but with locally increased contrast and denser aggregation, reflecting the additional presence of Ag nanoparticles at the CN560/BiOI interface. These Ag domains act as conductive bridges that further reinforce interfacial connectivity without disrupting the underlying two‐dimensional diffusion pathways of either semiconductor. Elemental mapping by TEM–EDS and SEM–EDS (Figures S14, S15) confirms the uniform spatial distribution of C, N, Bi, O, I, and Ag throughout the composite, demonstrating that the ball‐milling strategy yields chemically homogeneous materials with continuous and well‐integrated interfaces.

Collectively, the TEM observations establish that mechanochemical processing preserves the intrinsic layered structures of CN560 and BiOI while generating dense, electronically accessible heterojunctions. The combination of structural integrity and intimate nanoscale contact provides a physical basis for efficient charge separation and transport, which underpins the enhanced photocatalytic stability and performance discussed in subsequent sections.

**FIGURE 3:**
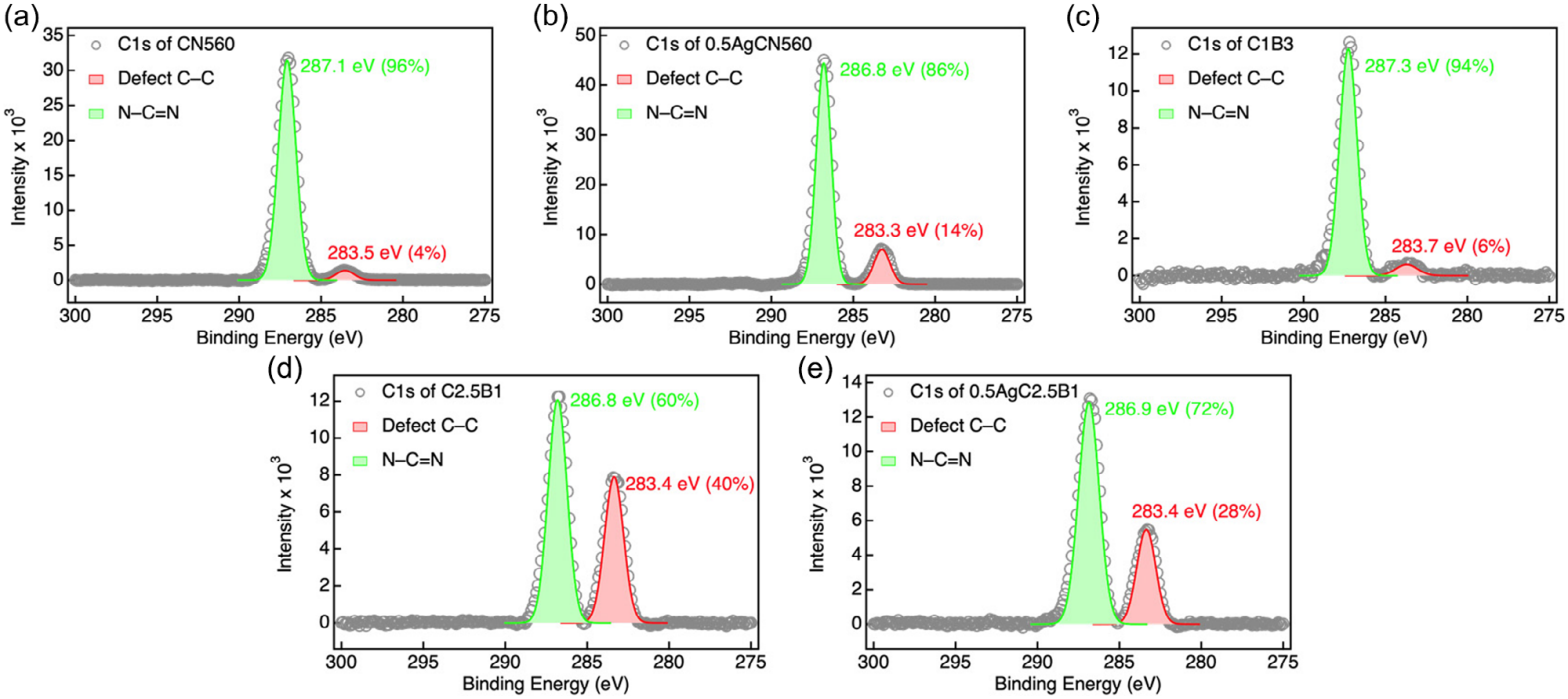
High‐resolution C 1s XPS spectra of (a) CN560, (b) 0.5AgCN560, (c) C1B3, (d) C2.5B1, and (e) 0.5AgC2.5B1, deconvoluted using Gaussian functions. XPS = X‐ray photoelectron spectroscopy.

High‐resolution X‐ray photoelectron spectroscopy (XPS) measurements provide atomic‐scale insight into the evolution of chemical bonding and electronic environments across the heterojunction interfaces (Figure 3). For pristine CN560, the C 1s spectrum is dominated by the N–C=N component at 287.1 eV, accompanied by a minor C–C contribution at 283.5 eV associated with structural defects [41, 42]. After Ag photodeposition, the relative intensity of the C–C component increases from 4% to 14%, indicating electronic perturbation of the conjugated heptazine framework via weak interfacial interaction with metallic Ag rather than formation of new covalent bonds. Consistently, Fourier‐transform infrared spectroscopy (FT‐IR, Figure S16) shows that the characteristic vibrational features of *g*‐C_3_N_4_, including NH stretching (3300–3000 cm^−1^), C=N and C–N vibrations (1628–1200 cm^−1^), and the triazine ring breathing mode at 805 cm^−1^, remain unchanged [43], confirming preservation of the *g*‐C_3_N_4_ backbone upon Ag photoreduction.

The N 1s spectra (Figure S17) further support this interpretation. Three components corresponding to C–N=C, N–(C)_3_, and NH_*x*_ species are observed [44]. No significant shift is detected after Ag deposition, whereas the hydrothermal composite C1B3 exhibits a pronounced increase in NH_*x*_ intensity, indicative of partial protonation at the CN/BiOI interface. In contrast, the ball‐milled C2.5B1 sample shows only a subtle NH_*x*_ increase, suggesting that mechanochemical treatment induces interfacial rearrangement without extensive chemical degradation of the *g*‐C_3_N_4_ framework.

In the Bi 4f region (Figure S18), both hydrothermal and ball‐milled composites display pronounced binding‐energy shifts (≈2 and 3 eV, respectively) relative to pristine BiOI, accompanied by complete disappearance of the Bi 4f satellite features [45]. Such large shifts exceed those typically associated with simple band bending and instead indicate substantial modification of the local chemical environment and orbital screening of Bi species induced by strong interfacial coupling with *g*‐C_3_N_4_. This interpretation is reinforced by parallel changes in the O 1s spectra (Figure S19), where a new component emerges at ≈529 eV and is assigned to interfacial C–O or C = O species formed during bond reconstruction. The I 3d spectra (Figure S20) exhibit a similar evolution. Characteristic satellite features present in pristine BiOI gradually weaken in the hydrothermal composite and vanish entirely in the ball‐milled C2.5B1 sample, reflecting significant alteration of the electronic screening and coordination environment of iodide species within the heterostructure.

Additional insight into interfacial electronic redistribution is obtained from the Ag 3d spectra (Figure S21) [39]. The Ag 3d doublet positions are consistent with predominantly metallic Ag0, and no features corresponding to oxidized Ag species are detected. Upon formation of the indirect Z‐scheme composite 0.5AgC2.5B1, the Ag 3d5/2 peak shifts positively by ≈0.4 eV, indicating that Ag resides in an electron‐deficient environment after interfacial contact. Importantly, this binding‐energy shift reflects Fermi‐level equilibration among CN560, Ag, and BiOI under dark conditions, rather than photoinduced carrier migration. The resulting dark‐state electronic redistribution establishes an internal electric field at the interfaces, which subsequently governs the direction of photoexcited charge transfer discussed in the mechanistic section below. High‐resolution XPS analysis of the indirect Z‐scheme sample (Figure S22) further confirms that the core‐level profiles of Bi, O, and I remain nearly identical to those of the direct Z‐scheme composite, indicating preservation of bulk composition and highlighting the interface‐specific nature of the electronic modifications mediated by Ag nanoparticles.

**FIGURE 4:**
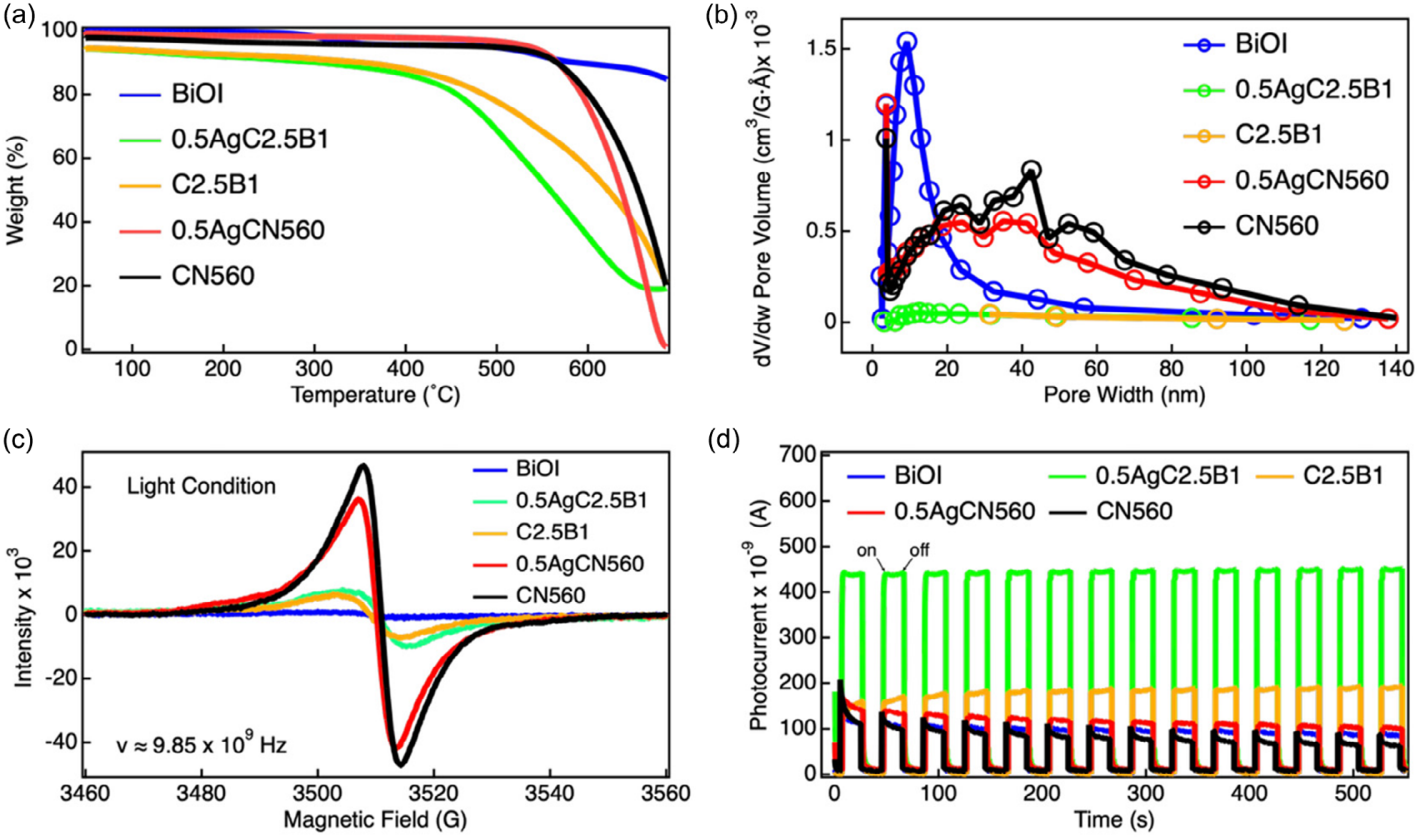
(a) TGA curves of CN560, 0.5AgCN560, C2.5B1, 0.5AgC2.5B1, and BiOI. (b) N_2_ adsorption–desorption isotherms and pore size distributions from BET analysis. (c) EPR spectra of the five samples recorded at 9.85 GHz. (d) Transient photocurrent responses under intermittent light irradiation. BET = Brunauer–Emmett–Teller; EPR = electron paramagnetic resonance; TGA = thermogravimetric analysis.

**FIGURE 5:**
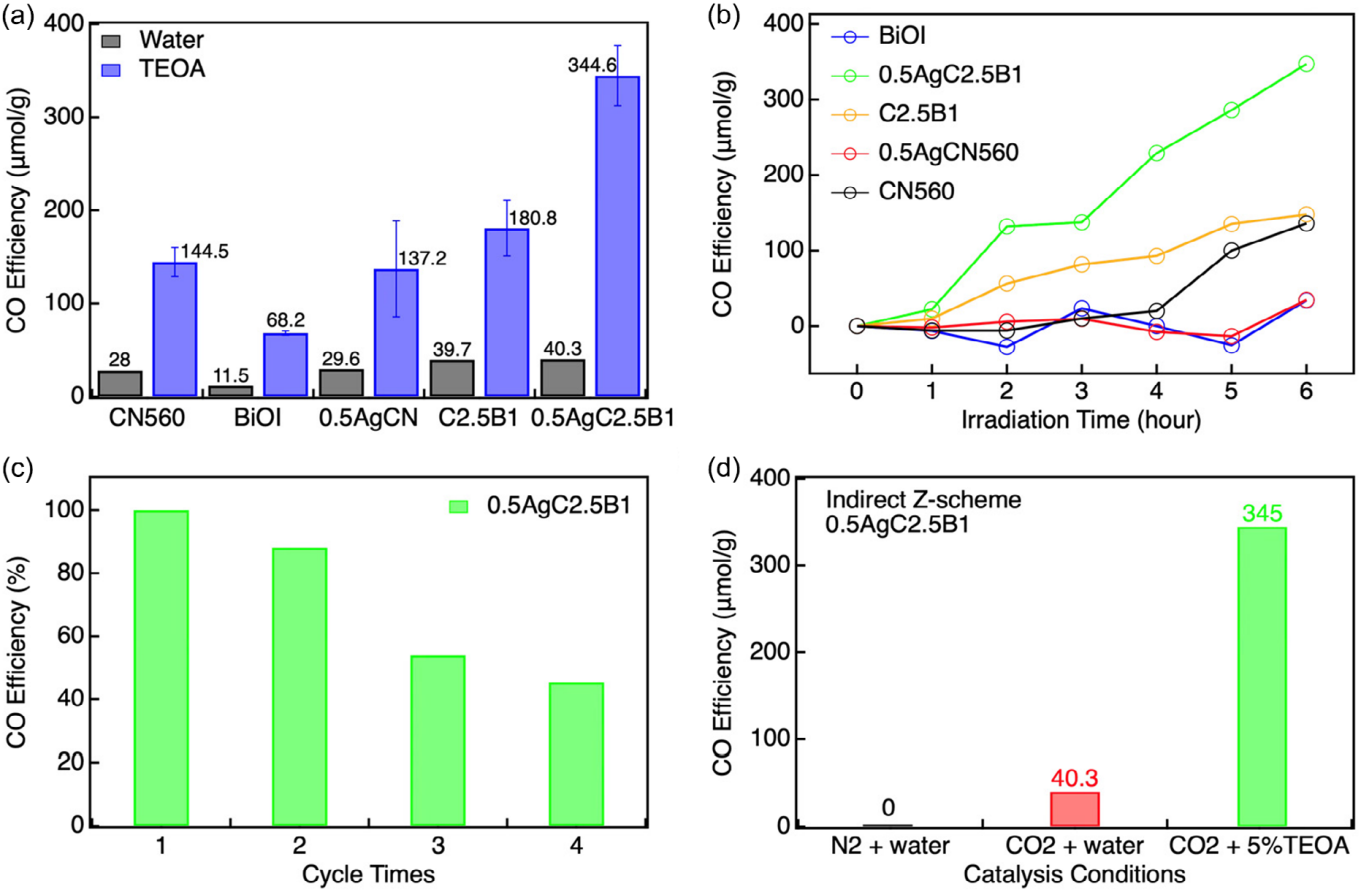
(a) CO yields from photocatalytic CO_2_ reduction over CN560, 0.5AgCN560, C2.5B1, 0.5AgC2.5B1, and BiOI, tested in both water and triethanolamine (TEOA) environments (background CO from TEOA photolysis has been subtracted) for 6 h. (b) Time‐resolved CO production profiles during 6 h irradiation corrected by subtracting the CO background arising from TEOA photolysis, as determined from blank experiments. (c) Cycling stability tests of 0.5AgC2.5B1 over four runs. (d) Control experiments under N_2_ and CO_2_ atmospheres.

Collectively, the integrated XPS and FT‐IR analyses demonstrate that both hydrothermal and mechanochemical routes successfully construct Z‐scheme heterojunctions, while the ball‐milling strategy yields more homogeneous interfacial bonding and stronger electronic coupling. The correlated shifts in the Bi 4f, O 1s, and I 3d regions, together with satellite‐peak suppression and the emergence of interfacial C–O species, provide compelling evidence for pronounced interfacial reconstruction rather than simple band bending or framework degradation. These chemically coupled interfaces establish a structural and electronic basis for efficient charge redistribution, consistent with the enhanced photocurrent responses and attenuated electron paramagnetic resonance (EPR) signals shown in Figure 4, and they ultimately account for the superior CO_2_ reduction performance shown in Figure 5.

Thermogravimetric analysis (TGA, Figure 4a; see also Figure S23) reveals distinct degradation behaviors among the pristine and composite samples, providing insight into how interfacial coupling influences framework robustness. Pristine CN560 decomposes at ≈611°C with a total weight loss of 76% by 685°C, corresponding to the collapse of the heptazine backbone. In contrast, BiOI exhibits a two‐step degradation process at 269°C and 504°C, associated with sequential phase transformations to Bi_4_O_5_I_2_, Bi_5_O_7_I, and Bi_2_O_3_ [46]. Upon Ag photodeposition, CN560 shows a slightly reduced decomposition onset temperature (597°C) and an increased mass loss (94%), consistent with partial perturbation of N–C=N bonding environments observed in the C 1s and N 1s XPS spectra (Figure 3). The ball‐milled composites exhibit further decreases in thermal stability (C2.5B1: 570°C; 0.5AgC2.5B1: 444°C), reflecting interfacial chemical reconstruction induced by mechanochemical treatment.

It should be emphasized that these changes in decomposition temperature reflect high‐temperature thermal stability rather than operational stability under photocatalytic conditions. All CO_2_ reduction reactions in this study were conducted at near‐ambient temperature without external heating, far below the onset of thermal decomposition observed by TGA. Therefore, the reduced thermal stability primarily signifies modified bonding environments and enhanced interfacial coupling rather than a tendency toward structural degradation during photocatalytic operation. Consistently, the indirect Z‐scheme composite 0.5AgC2.5B1 exhibits stable activity over repeated photocatalytic cycles (Figure 5c), confirming the preservation of structural integrity under prolonged illumination.

Nitrogen adsorption–desorption measurements (Figure 4b; see also Figure S24) highlight a pronounced trade‐off between surface porosity and interfacial compactness. Pristine CN560 and BiOI display relatively high Brunauer–Emmett–Teller (BET) surface areas (61 and 59 m^2^ g^−1^, respectively), characteristic of their lamellar architectures. Incorporation of Ag nanoparticles moderately reduces the surface area of CN560 to 51 m^2^ g^−1^ due to partial pore occupation. In contrast, both ball‐milled Z‐scheme composites exhibit drastically reduced surface areas (C2.5B1: 5.6 m^2^ g^−1^; 0.5AgC2.5B1: 6.1 m^2^ g^−1^) with narrowed pore‐size distributions. This substantial decrease arises from collapse of interparticle voids and densification of stacked nanosheets induced by mechanochemical processing rather than amorphization of the crystal lattice. Supporting this interpretation, XRD analysis (Figure 1b) confirms retention of the characteristic (002) reflection of *g*‐C_3_N_4_, indicating preservation of the layered framework despite morphological compaction. Although such densification markedly reduces the accessible surface area, it simultaneously enhances interfacial contact and electronic coupling between CN560 and BiOI domains. In this system, photocatalytic CO_2_ reduction is governed primarily by charge separation and interfacial charge transfer rather than by the total geometric surface area.

EPR (Figure 4c; see also Figure S25) provides complementary insight into the behavior of unpaired electrons associated with the heptazine motifs of *g*‐C_3_N_4_. All samples exhibit a signal at g ≈2.004, characteristic of nitrogen‐centered radicals. Pristine CN560 shows the strongest signal intensity, reflecting abundant unpaired electrons confined within the conjugated π‐system. Upon Ag photodeposition and subsequent composite formation, the EPR intensity decreases markedly. This attenuation can be attributed to partial delocalization or trapping of electrons at metallic Ag sites, as well as to interfacial chemical reconstruction that modifies the local electronic environment of the heptazine units. Importantly, XPS and FT‐IR analyses reveal local bond reorganization and formation of interfacial C–O species rather than complete framework collapse, while the persistence of the (002) XRD reflection confirms retention of the long‐range layered structure. In the direct Z‐scheme composite C2.5B1, the EPR signal is further weakened, consistent with enhanced interfacial charge redistribution and recombination. The indirect Z‐scheme sample 0.5AgC2.5B1 exhibits a comparably attenuated signal, indicating efficient spatial separation and stabilization of charge carriers within the heterostructure. This behavior, together with the enhanced photocurrent response (Figure 4d), supports a scenario in which interfacial electronic coupling and chemical reconstruction jointly suppress bulk recombination and prolong carrier lifetimes.

Transient photocurrent measurements (Figure 4d) directly probe charge separation and transport dynamics. Pristine CN560 exhibits a high initial photocurrent spike followed by pronounced decay under continuous illumination, with progressive loss of peak intensity over repeated cycles, indicative of severe recombination and defect‐dominated charge trapping. BiOI and 0.5AgCN560 show less severe decay but still display gradual attenuation of peak photocurrent, suggesting limited interfacial stability. In contrast, the direct Z‐scheme composite C2.5B1 shows moderate enhancement accompanied by a gradual increase in photocurrent during the initial cycles, revealing an induction process associated with surface activation, trap‐state filling, and interfacial equilibration with the electrolyte. After this activation stage, the photocurrent stabilizes without evidence of irreversible degradation. Notably, the indirect Z‐scheme composite 0.5AgC2.5B1 delivers the highest photocurrent density and maintains a nearly constant response over repeated on/off cycles, demonstrating both efficient carrier separation and excellent operational stability. This superior photoresponse confirms that the Ag‐mediated charge transfer pathway effectively suppresses interfacial recombination and facilitates directional charge transport across the CN560/BiOI heterojunction.

Taken together, the TGA, BET, EPR, and photocurrent analyses establish a coherent structure property relationship. Mechanochemical ball milling produces densely bonded heterojunctions with reduced porosity but substantially enhanced interfacial contact and electronic coupling, while Ag nanoparticles function as conductive mediators that sustain directional charge transfer. Although the accessible surface area is drastically reduced, the compact architecture shortens carrier diffusion distances and forms continuous charge transport pathways across the CN560/BiOI interface. Consequently, the enhanced photocatalytic activity of 0.5AgC2.5B1 originates from improved interfacial electronic connectivity and charge transfer efficiency rather than from an increased density of surface adsorption sites, providing a rational explanation for the performance trends shown in Figure 5.

The photocatalytic CO_2_ reduction activities of all samples were systematically evaluated in both pure water and triethanolamine (TEOA) environments. Figure 5a summarizes the CO yields obtained after 6 h of irradiation, while additional datasets collected under extended 12 h illumination is provided in the SI (Tables S6–S9). In pure water, pristine CN560 produces 28 μmol g^−1^ of CO after 6 h, whereas BiOI is nearly inactive (11.5 μmol g^−1^) due to its conduction band edge being insufficiently negative to drive CO_2_/CO reduction. Notably, the CO yields of CN560 and 0.5AgCN560 are comparable within experimental uncertainty (144.5 ± 16 vs. 137.2 ± 52 μmol g^−1^), indicating that Ag deposition on *g*‐C_3_N_4_ alone does not lead to a statistically significant enhancement under the present conditions. This observation suggests that metallic Ag may introduce competing effects, such as partial surface coverage, light shielding, or formation of interfacial recombination sites, which offset any potential benefit associated with modified optical absorption. Accordingly, although Ag introduces a visible‐light absorption feature, the photocatalytic performance data indicate that a plasmonic hot‐electron contribution, if present, is not the dominant factor governing the reaction rate. Instead, the functional role of Ag becomes pronounced only when it participates in interfacial charge transfer mediation within a heterojunction architecture. The influence of Ag loading was further examined under extended irradiation (Figure S26). After 12 h, pristine CN560 produces 32.8 μmol g^−1^ of CO, whereas the 0.5 wt% Ag‐loaded sample reaches 93.6 μmol g^−1^, suggesting that moderately dispersed Ag nanoparticles can exert a positive effect under prolonged illumination. This time‐dependent enhancement can be attributed to the gradual suppression of charge recombination by Ag nanoparticles, which act as hole sinks and facilitate charge separation. While the effect is less pronounced at shorter irradiation times, it becomes increasingly significant under prolonged illumination, leading to enhanced cumulative CO production. Higher Ag loadings (1.0–1.5 wt%) result in decreased activity (39.2–33.2 μmol g^−1^), consistent with aggregation or surface shielding effects. Importantly, the optimum at 0.5 wt% Ag is consistent across both 6  and 12 hr datasets, despite differences in absolute yields.

The formation of direct Z‐scheme heterojunctions significantly improves catalytic efficiency. The hydrothermally synthesized C1B3 composite reaches 80.4 μmol g^−1^ after 12 h, while the ball‐milled C2.5B1 achieves 38.3 μmol g^−1^ after only 6 h irradiation (Figures S27,S28; Tables S8, S9). Although the hydrothermal route delivers higher instantaneous yields, its large standard deviation reflects poor reproducibility. This variability likely arises from sedimentation of *g*‐C_3_N_4_, sensitivity of BiOI nucleation to local concentration gradients, and nonuniform interfacial contact. In contrast, the ball‐milled C2.5B1 composite exhibits smaller deviations and consistent activity, confirming more effective and reproducible heterojunction formation. To explicitly assess the necessity of mechanochemical treatment, a *g*‐C_3_N_4_/BiOI composite with the same weight ratio (2.5:1) was prepared by simple physical mixing and tested under identical conditions. As shown in Figure S29, the hand‐mixed sample yields only 13.8 μmol g^−1^ of CO, markedly lower than the ball‐milled C2.5B1 (39.7 μmol g^−1^). This result demonstrates that intimate interfacial contact, rather than simple coexistence of the two components, is essential for efficient charge transfer. Among all systems, the indirect Z‐scheme composite 0.5AgC2.5B1 exhibits the highest activity in pure water, producing 40.3 μmol g^−1^ of CO despite its reduced surface area (Figure 4b). Here, Ag nanoparticles act as efficient electron mediators that compensate for structural densification by facilitating directional charge transport between CN560 and BiOI domains. Upon introduction of TEOA as a sacrificial electron donor, the CO yield of 0.5AgC2.5B1 increases dramatically to 344.6 μmol g^−1^ (57.4 μmol g^−1^ h^−1^) after 6 h irradiation, nearly eightfold higher than that in water and substantially exceeding all other samples. This pronounced enhancement highlights the synergistic effect of Ag‐mediated charge transfer within the indirect Z‐scheme architecture. To further evaluate the role of surface area, the CO yield was normalized by the BET surface area. The resulting activities are 2.37, 1.16, 2.69, 32.29, and 56.49 μmol m^−2^ for CN560, BiOI, 0.5AgCN560, C2.5B1, and 0.5AgC2.5B1, respectively. Notably, the normalized activity of 0.5AgC2.5B1 is ≈24 times higher than that of CN560, despite its significantly reduced surface area. These results suggest that photocatalytic CO_2_ reduction in this system is not governed by the total accessible surface area, but rather by interfacial electronic effects. Although BET surface area does not directly reflect the number of active sites, the pronounced increase in normalized activity highlights the dominant role of interfacial charge transfer processes.

Time‐resolved CO evolution profiles (Figure 5b) further demonstrate the kinetic superiority of the indirect configuration. CN560 and BiOI exhibit negligible CO formation during the first 4 h (≈3 and 0 μmol g^−1^ h^−1^, respectively), whereas the direct Z‐scheme C2.5B1 reaches a rate of 24.6 μmol g^−1^ h^−1^. In contrast, 0.5AgC2.5B1 achieves 53.2 μmol g^−1^ h^−1^, more than twice that of the direct Z‐scheme counterpart. To exclude contributions from TEOA photodecomposition, blank experiments were conducted under identical conditions without photocatalyst (Figure S30). The CO generated from TEOA photolysis was quantified as a function of irradiation time and subtracted from the total CO detected in the presence of catalysts. The corrected CO evolution curves in Figure 5b therefore reflect only the intrinsic catalytic contribution of each material, with all TEOA‐derived artifacts rigorously removed.

Recycling tests (Figure 5c) show that 0.5AgC2.5B1 retains ≈45% of its initial activity after four consecutive runs, suggesting partial surface fouling or gradual depletion of sacrificial donor as plausible deactivation pathways. In Figure 5d, all photocatalytic reactions were carried out under continuous light irradiation (AM 1.5G, 100 mW cm^−2^). The “N_2_ + water” and “CO_2_ + water” conditions refer to photocatalytic systems without sacrificial agents, rather than dark control experiments, while “CO_2_ + 5% TEOA” represents a system with a sacrificial electron donor. Control experiments under N_2_ atmosphere (Figure 5d), performed under identical illumination conditions, show no detectable CO formation, confirming that CO originates exclusively from CO_2_ reduction rather than from catalyst decomposition, contamination, or thermal effects. Under these conditions, CO_2_ serves as the reduction reactant, while H_2_O participates in the oxidation half‐reaction. A measurable CO yield (40.3 μmol·g^−1^) is therefore attributed to photocatalytic CO_2_ reduction.

Collectively, these results establish the indirect Z‐scheme 0.5AgC2.5B1 as the most effective configuration for photocatalytic CO_2_ reduction in this system. By integrating a conductive Ag bridge with a compact and electronically coupled CN560/BiOI interface, this architecture simultaneously achieves enhanced charge separation, improved kinetics, and superior reproducibility. The findings validate the rational design of indirect Z‐scheme heterojunctions as a viable strategy for efficient and controllable solar‐driven CO_2_ conversion.

**FIGURE 6:**
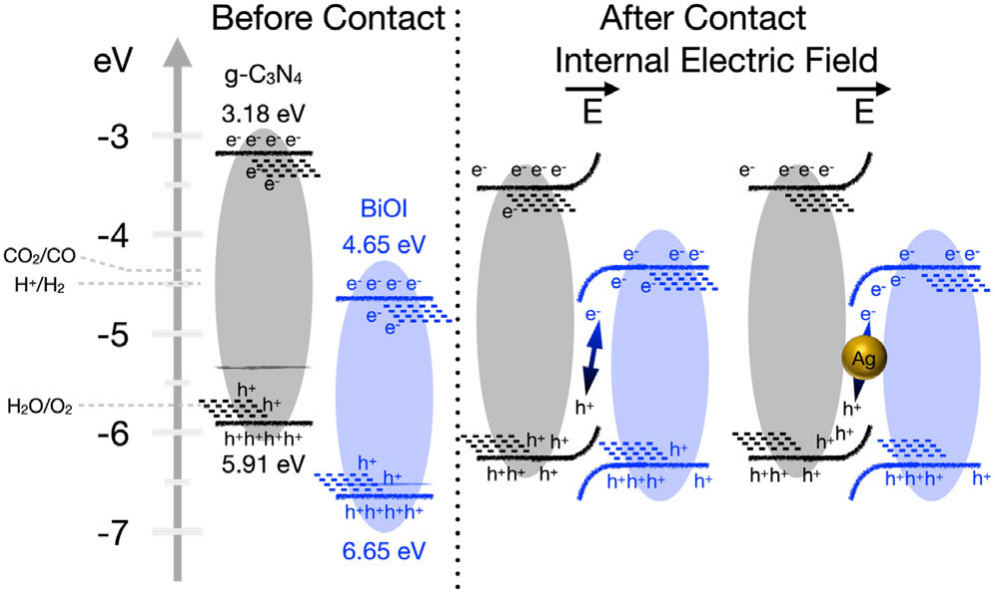
Schematic energy‐band alignment and charge transfer pathways of the direct and indirect Z‐scheme heterojunctions constructed from *g*‐C_3_N_4_, BiOI, and Ag nanoparticles, derived from UPS measurements and optical bandgap analysis.

On the basis of ultraviolet photoelectron spectroscopy (UPS, Figure S31) combined with optical bandgap analysis (Figure 1d), the energy‐band alignments of CN560, BiOI, and their heterostructures are shown in Figure 6. Pristine CN560 exhibits a conduction band minimum positioned at a sufficiently negative potential to thermodynamically drive CO_2_ reduction, whereas BiOI possesses a relatively deep valence band maximum that favors oxidative half‐reactions. Importantly, these band positions also allow a clear distinction between a Type‐II heterojunction and a Z‐scheme configuration.

In a conventional Type‐II alignment, photoexcited electrons would transfer from the CB of CN560 to the CB of BiOI, while holes migrate in the opposite direction. However, the CB of BiOI lies below the CO_2_/CO redox potential, rendering such transferred electrons incapable of driving CO_2_ reduction. This scenario is therefore inconsistent with the experimentally observed CO evolution. Instead, the UPS‐derived band alignment supports a Z‐scheme charge transfer pathway, in which photoexcited electrons in the CB of BiOI recombine with holes in the VB of CN560 at the interface. This interfacial recombination selectively removes low‐energy charge carriers while preserving high‐energy electrons in CN560 and strongly oxidative holes in BiOI, which, respectively, drive the CO_2_‐to‐CO reduction and water oxidation half‐reactions. The lattice contraction observed in XRD (Figure 1b) and the disappearance of Bi 4f satellite features in XPS (Figures S18 and S19) further substantiate strong electronic coupling at the CN560/BiOI interface, consistent with a Z‐scheme rather than a Type‐II heterojunction.

In contrast, the indirect Z‐scheme heterojunction (0.5AgC2.5B1) incorporates metallic Ag nanoparticles as conductive electron mediators with an intermediate work function between CN560 and BiOI. It is important to emphasize that the binding‐energy shifts observed in XPS represent electronic equilibration in the dark state, arising from Fermi‐level alignment among CN560, Ag, and BiOI, rather than photoinduced carrier migration. This dark‐state charge redistribution establishes an internal electric field across the interfaces, which dictates the subsequent light‐driven charge transfer behavior. Upon illumination, photoexcited electrons in the CB of BiOI migrate into the Ag domains and recombine with holes from the VB of CN560 at the Ag surface. This Ag‐mediated recombination pathway spatially separates the remaining charge carriers, preserving high‐energy electrons in CN560 and deep‐lying holes in BiOI. As a result, bulk recombination in both semiconductors is effectively suppressed.

The positive shift of the Ag 3d binding energy (Figure S21), together with the consistent trends observed in the Bi, O, and I core‐level spectra (Figure S22), confirms interfacial electron redistribution mediated by the Ag bridge. Functional evidence for this charge transfer pathway is further provided by the enhanced photocurrent responses (Figure 4d) and attenuated EPR signals (Figure 4c), which collectively indicate more efficient charge separation and prolonged carrier lifetimes in the indirect Z‐scheme system.

Taken all together, these results establish a coherent mechanistic framework. The direct Z‐scheme relies on intimate CN560/BiOI contact to enable interface‐localized recombination, whereas the indirect Z‐scheme introduces a metallic Ag bridge that constructs a spatially extended and energetically favorable charge transfer network. This Ag‐mediated architecture minimizes recombination losses while maintaining strong redox potentials, thereby accounting for the superior activity and stability of 0.5AgC2.5B1 shown in Figure 5. The findings validate the rational design of Ag‐bridged indirect Z‐scheme heterostructures as an effective strategy for solar‐driven CO_2_ conversion.

A concise performance comparison between this work and representative *g*‐C_3_N_4_‐based photocatalysts reported in the literature is shown in Table S10. It is acknowledged that indirect Z‐scheme systems and Ag‐mediated heterojunctions have been previously reported. In many of these studies, however, the indirect charge transfer nature was identified a posteriori, with the underlying mechanism inferred only after experimental observation of enhanced charge separation or catalytic performance.

In contrast, the present study adopts a design‐driven strategy in which Ag nanoparticles are deliberately introduced from the outset as an interfacial bridge, guided by their work function being positioned between those of *g*‐C_3_N_4_ and BiOI. This band‐engineering‐based approach enables controlled construction of a metal‐bridged indirect Z‐scheme architecture, rather than relying on post hoc mechanistic interpretation. Moreover, by constructing both direct and indirect Z‐scheme heterojunctions from the same semiconductor pair under identical reaction conditions, this work establishes an architecture‐controlled comparison that explicitly isolates the role of the charge transfer pathway and the metal mediator. Such a systematic comparative framework is rarely implemented in previous studies and provides a clear experimental basis for distinguishing interfacial charge transfer mechanisms within a well‐defined model system. Accordingly, the primary contribution of this work does not lie in introducing a new material combination, but in demonstrating how interfacial energetics and charge transfer routes can be rationally designed, controlled, and experimentally differentiated in photocatalytic CO_2_ reduction systems.

## 3 Conclusions

In summary, a rational and solvent‐free strategy has been developed to construct both direct and indirect Z‐scheme heterojunctions by integrating *g*‐C_3_N_4_, BiOI, and Ag nanoparticles through mechanochemical ball milling and subsequent photodeposition for photocatalytic CO_2_ reduction. Comprehensive structural and spectroscopic analyses, including XRD, TEM, XPS, EPR, and UPS, demonstrate that these processes induce intimate interfacial coupling and interfacial chemical reconstruction, leading to pronounced charge redistribution across the heterojunction interfaces. Among all investigated systems, the indirect Z‐scheme composite 0.5AgC2.5B1 exhibits the highest photocatalytic performance, delivering a CO evolution of 344.6 μmol g^−1^ after 6 h of irradiation, markedly surpassing pristine CN560, BiOI, and the corresponding direct Z‐scheme composite. This enhancement originates from the Ag‐mediated charge transfer pathway, in which metallic Ag nanoparticles serve as efficient electron mediators that bridge CN560 and BiOI, suppress interfacial recombination, and promote directional carrier migration toward catalytically active sites. Mechanistic investigations reveal that the intermediate work function of Ag enables effective spatial separation of photogenerated charge carriers, preserving the strong reduction capability of CN560 and the oxidative potential of BiOI. The realization of this indirect Z‐scheme mechanism is consistently supported by UPS‐derived band alignment, XPS core‐level shifts, suppressed EPR signals, and enhanced photocurrent responses. Overall, this work demonstrates that mediator‐assisted interfacial engineering provides a robust and controllable route to regulate charge transfer pathways in *g*‐C_3_N_4_‐based heterojunctions. Beyond achieving superior photocatalytic CO_2_ reduction performance compared with conventional direct Z‐scheme systems, the design strategy presented here establishes a generalizable framework for tuning interfacial energetics and charge transport architectures in advanced photocatalysts for solar‐to‐chemical energy conversion.

## 4 Experimental Section

4.1

Details of chemicals, catalyst synthesis, characterizations, supplementary figures and tables are provided in SI.

## Supporting Information

Additional supporting information can be found online in the Supporting Information section.

## Funding

This study was supported by National Science and Technology Council (NSTC 113‐2639‐M‐A49‐001‐ASP, NSTC 114‐2639‐M‐A49‐001‐ASP).

## Conflicts of Interest

The authors declare no conflicts of interest.

## Supporting information

Supplementary Material

## Data Availability

The data that support the findings of this study are available on request from the corresponding author. The data are not publicly available due to privacy or ethical restrictions.
