# Supplementary material for: Enhanced Photocatalytic Performance for CO2 Reduction Using an Indirect Z‐Scheme Heterojunction Photocatalyst
Source: ChemSusChem. 2026 May 12;19(9):e70716. doi: 10.1002/cssc.70716 (PMC13168366; doi:10.1002/cssc.70716)
Supplement: Supplementary file 1 — Supplementary Material [file CSSC-19-e70716-s001.pdf]

Enhanced Photocatalytic Performance for CO_2 Reduction Using an Indirect Z-scheme Heterojunction Photocatalyst

*I-Hua Tsai[1], Chen-Hsiu Fu[1], Ting-Hui Lin[1], Shu-Yu Lin[1], and Eric Wei-Guang Diau[1, 2, *]*

[1]Department of Applied Chemistry, Institute of Molecular Science, National Yang Ming Chiao Tung University, 1001 Ta-Hseuh Rd., Hsinchu 300093, Taiwan

[2]Center for Emergent Functional Matter Science, National Yang Ming Chiao Tung University, 1001 Ta-Hseuh Rd., Hsinchu 300093, Taiwan

[*]Correspondence: diau@nycu.edu.tw

Experimental Section

Chemicals and Materials

Thiourea (≥99%, Sigma-Aldrich), bismuth nitrate pentahydrate ($Bi(NO_3)_3 \cdot 5H_2O$, 98%, Thermo Scientific), potassium iodide (KI, ≥99%, Sigma-Aldrich), silver nitrate ($AgNO_3$, ≥99.8%, Sigma-Aldrich), sodium citrate tribasic dihydrate (≥99%, Sigma-Aldrich), triethanolamine (TEOA, ≥99%, Sigma-Aldrich), hydrochloric acid (HCl, 37%, Honeywell Fluka), nitric acid (HNO_3, 70%, Honeywell Fluka), ethylene glycol (EG, 99%, J.T. Baker), ethanol (99.5%), and deionized (DI) water (18.2 MΩ·cm) were used in this study. Gases including CO_2, N_2, He, and zero-grade air (all 99.99%, Hsin Da Gas Co., Taiwan) were employed for photocatalytic reactions and characterization. All chemicals were used as received without further purification.

Instrumentation and Characterizations

A planetary ball mill (PM-100, Retsch) equipped with zirconia (ZrO_2) jars and 3 mm zirconia balls was used for mechanochemical synthesis. A high-temperature furnace (DF-20/MF-20, DENG YNG), vacuum oven (DOV-30, DENG YNG), ultrasonic cleaner (Easy 30H, Elma), and centrifuge (Model 3740, Kubota) were

employed for sample preparation. Photocatalytic tests were carried out under simulated solar irradiation using a solar simulator (XES-502S, San-Ei Electric) with an AM 1.5G filter and a light intensity of 100 mW·cm^{-2}. The products were characterized by powder X-ray diffraction (XRD, D8 Advance, Bruker) with Cu Kα radiation (λ=1.5406 Å), UV–vis diffuse reflectance spectroscopy (UV–vis DRS, V-760, JASCO), X-ray photoelectron spectroscopy (XPS) and ultraviolet photoelectron spectroscopy (UPS, ESCALAB Xi^{+}, Thermo Scientific), scanning electron microscopy (SEM, JSM-7401F, JEOL), transmission electron microscopy (TEM, JEM-F200, JEOL), Fourier transform infrared spectroscopy (FT-IR, FT/IR-6100, JASCO), thermogravimetric analysis (TGA, SDT 650, TA Instruments), electron paramagnetic resonance (EPR, BRUKER, ELEXSYS, E-580) and Brunauer–Emmett–Teller (BET) surface area analysis (ASAP 2460, Micromeritics).

Synthesis of Graphitic Carbon Nitride (g-C_3N_4)

Graphitic carbon nitride (g-C_3N_4) was synthesized by the thermal condensation of thiourea.[1] In a typical procedure, 5.0 g of thiourea was placed in a covered ceramic crucible and calcined in a muffle furnace under ambient air. The sample was heated from room temperature (~20 °C) to the target temperature (540, 560, or 580 °C) at a ramping rate of 2.25 °C·min^{-1}, followed by isothermal treatment for 4 h. After completion of the reaction, the furnace was allowed to cool naturally to room temperature. The resulting yellowish solid was collected and ground into a fine powder for subsequent use.

Photodeposition of Ag Nanoparticles on g-C_3N_4

Silver nanoparticles (AgNPs) were deposited on the surface of g-C_3N_4 through a light-driven reduction process, using silver nitrate as the Ag^{+} precursor and sodium citrate as the stabilizing agent.[2] Aqueous solutions of $AgNO_3$ and sodium citrate (10 mL each) were prepared independently, with $AgNO_3$ concentrations adjusted to yield Ag loadings of 0.3–1.5 wt% in the final photocatalysts. g-C_3N_4 powder (200 mg) was dispersed in a quartz photoreactor equipped with gas inlets/outlets, followed by the

sequential addition of the $AgNO_3$ and sodium citrate solutions, and then diluted to a total volume of 100 mL with deionized water. Prior to irradiation, the suspension was purged by alternate evacuation and N_2 purging (50 mL·min^{-1}) for six cycles to remove dissolved oxygen and establish an oxygen-free environment. Photodeposition was carried out under solar simulator with continuous stirring (300 rpm) for 1 h. During illumination, photogenerated electrons in g-C_3N_4 reduced Ag^+ to metallic Ag^0, leading to the formation of AgNPs on the g-C_3N_4 surface. After reaction, the suspension was centrifuged (12500 g, 5–10 min), and the precipitate was washed four times with a 1:1 (v/v) ethanol/deionized water mixture to remove residual reactants. The obtained solid was dried in a vacuum oven at 40 °C for 12 h, yielding Ag-modified g-C_3N_4 powders.

Hydrothermal Synthesis of BiOI

BiOI was synthesized via a hydrothermal method.[3] $Bi(NO_3)_3·5H_2O$ (3.0 mmol) and KI (3.0 mmol) were each dissolved in 30 mL of ethylene glycol, followed by ultrasonication for 15 min to ensure complete dissolution. The KI solution was added dropwise into the $Bi(NO_3)_3$ solution using a burette at a rate of 0.5 mL·min^{-1} under magnetic stirring (300 rpm). After complete addition, the mixture was stirred for an additional 1 h and then transferred into a 120 mL Teflon-lined stainless-steel autoclave. The sealed reactor was heated at 130 °C for 18 h in an oven. After naturally cooling to room temperature, the product was collected by centrifugation (12500 g, 5–10 min) and washed four times with deionized water to remove residual ions. The obtained solid was dried in a vacuum oven at 40 °C for 12 h, yielding uniform orange BiOI powders.

Hydrothermal Synthesis of g-C_3N_4/BiOI Composites

The g-C_3N_4/BiOI composites were prepared under the same hydrothermal conditions as pure BiOI. Briefly, after mixing the $Bi(NO_3)_3·5H_2O$ and KI solutions thoroughly, 200 mg of pre-calcined g-C_3N_4 powder was immediately added and dispersed by magnetic stirring (300 rpm) for 1 hour. The resulting suspension was transferred into a Teflon-lined stainless-steel autoclave and heated at 130 °C for 18 h. After cooling to room temperature, the products were separated by centrifugation

(12500 g, 5–10 min), washed four times with deionized water, and dried in a vacuum oven at 40 °C for 12 h to yield g-C_3N_4/BiOI composite powders.

Ball-Milling Synthesis of g-C_3N_4/BiOI Composites

g-C_3N_4/BiOI composites were also prepared by a solvent-free mechanochemical method using a planetary ball mill. Pre-calcined g-C_3N_4 and hydrothermally synthesized BiOI powders were weighed in the desired ratios and placed into a ZrO_2 milling jar with ZrO_2 balls. Ball milling was conducted at 350 rpm using a programmed milling cycle of 50 s on / 10 s off, with the rotation direction automatically reversed every 60 s to prevent shear-induced agglomeration and ensure uniform mixing. The total milling duration was 1 h.

After milling, 20–25 mL of deionized water was added to the jar, which was resealed and rotated at 400 rpm for 5 min (50 s on / 10 s off) to rinse adhered powders from the jar and balls. The resulting suspension was centrifuged (12500 g, 5–10 min), washed four times with deionized water, and dried in a vacuum oven at 40 °C for 12 h to obtain well-dispersed g-C_3N_4/BiOI composite powders.

Ball-Milling Synthesis of g-C_3N_4/Ag/BiOI Ternary Composites

The ternary g-C_3N_4/Ag/BiOI composites were prepared by a mechanochemical method. Ag/g-C_3N_4 powders obtained from the photodeposition step (Section 2.4) were mixed with hydrothermally synthesized BiOI in designated mass ratios and placed into a ZrO_2 milling jar with ZrO_2 balls. The milling was performed under the same conditions as described before (350 rpm, 50 s on / 10 s off, direction reversal once per minute) for a total of 1 h. The resulting powders were collected for subsequent characterization and photocatalytic tests.

Photocatalytic Reaction Setup

Photocatalytic CO_2 reduction reactions were carried out in a sealed quartz glass

reactor with a total volume of 180 mL. A predetermined amount of catalyst powder was evenly spread at the bottom of the reactor, followed by the addition of 2 mL of deionized water or an aqueous triethanolamine (TEOA) solution as the reaction medium. Prior to irradiation, the reactor was purged with high-purity N_2 (50 $mL \cdot min^{-1}$) for 10 min to remove dissolved oxygen. Subsequently, the gas flow was switched to high-purity CO_2 bubbled through a water-filled gas-washing bottle to ensure adequate humidity. CO_2 was continuously introduced at 50 $mL \cdot min^{-1}$ for 50 min to saturate the system atmosphere. Under these conditions, GC analysis confirmed that the reactor atmosphere was dominated by CO_2, with residual N_2 and O_2 accounting for less than 5% of the total gas composition. After confirming gas-tight sealing at all joints, the reactor was irradiated under solar simulator (AM 1.5G, 100 $mW \cdot cm^{-2}$) for 6 or 12 h, depending on the experimental conditions. During the reaction, the reactor atmosphere was maintained under a CO_2/H_2O mixture to drive the photocatalytic CO_2 reduction.

Gas Chromatography (GC) Analysis of Photocatalytic Products

After photocatalytic reactions, the evolved gases were immediately analyzed by gas chromatography to identify and quantify the products. Helium was used as the carrier gas at a flow rate of 10 $mL \cdot min^{-1}$. Gas separation was achieved using two different capillary columns: (i) 30 m length, 0.53 mm i.d., 50 μm film thickness (Thermo Scientific); and (ii) 30 m length, 0.53 mm i.d., 1 μm film thickness (Supelco). A thermal conductivity detector (TCD) was employed to detect inorganic gases (e.g., CO, H_2), while a flame ionization detector (FID) was used for hydrocarbons (e.g., CH_4, C_2H_4). The injector and detector temperatures were set to 100 and 200 °C, respectively, and the column temperature was maintained isothermally at 70 °C. Gas samples (1 mL) were withdrawn from the reactor using a gas-tight syringe (Hamilton) and injected into the GC system. Product peaks were identified by comparison with standard gases (CO, CH_4), and calibration curves were used to convert peak areas into molar concentrations. Product yields were reported in $\mu mol \cdot g^{-1}$ of catalyst. All measurements were repeated three times to ensure reproducibility and statistical reliability.

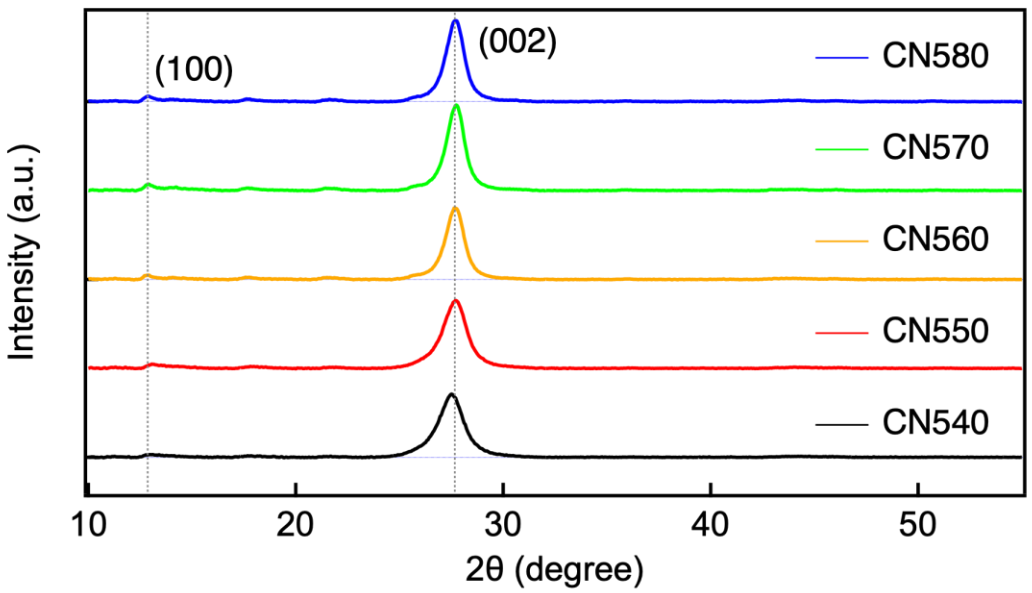

Figure S1. XRD patterns of g-C_3N_4 synthesized at different calcination temperatures (540–580 °C) using thiourea as a precursor.

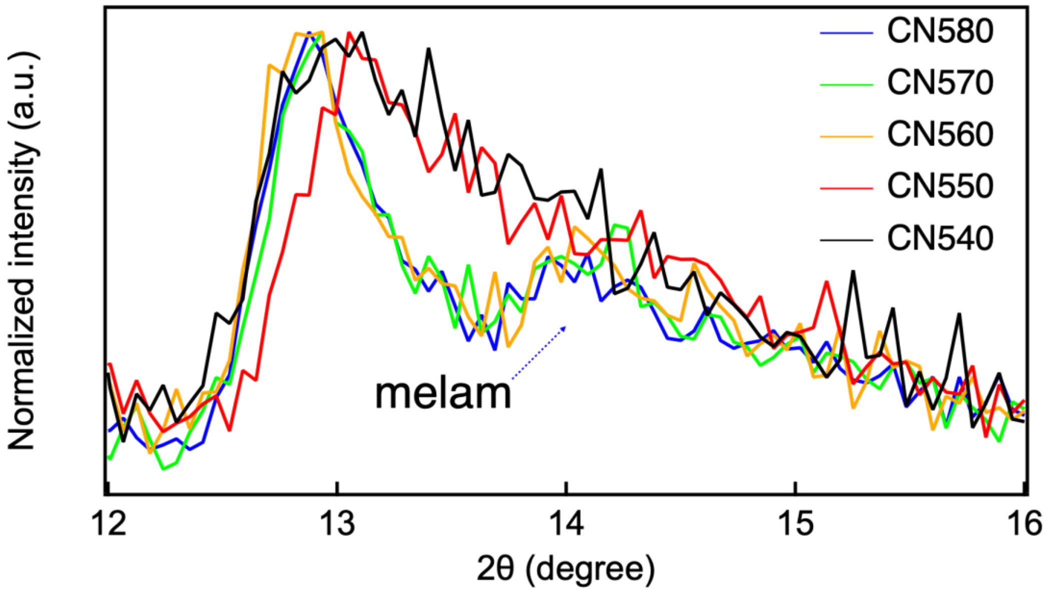

Figure S2. Enlarged and normalized XRD patterns focusing on the (100) reflection of g-C_3N_4 at different calcination temperatures.

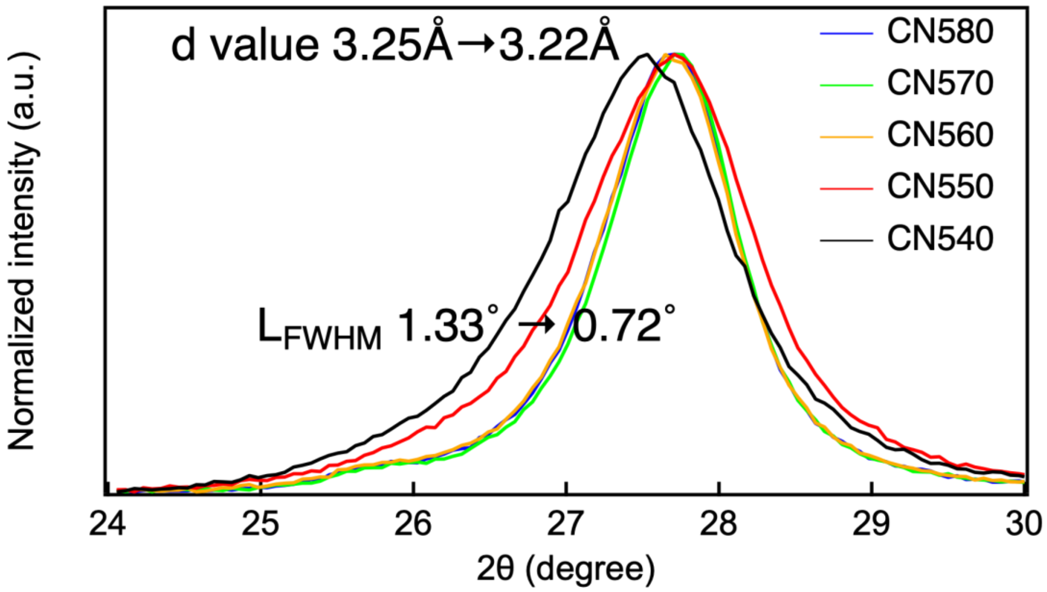

Figure S3. Enlarged and normalized XRD patterns of the (002) reflection of g-C_3N_4 at different calcination temperatures, fitted using the Voigt function (fitting parameters summarized in Table S1).

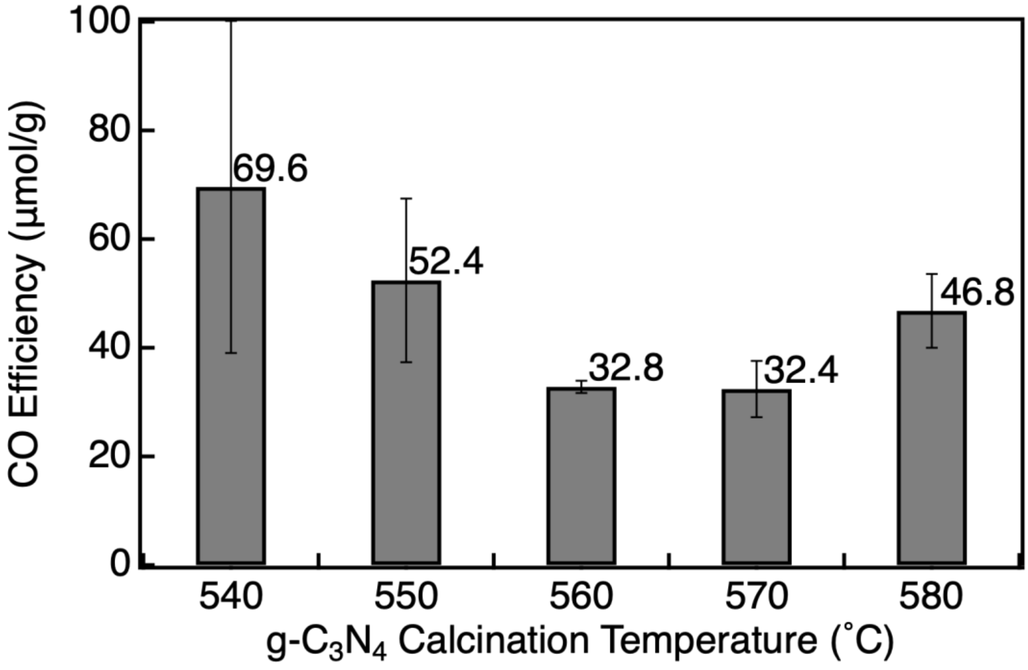

Figure S4. Photocatalytic CO_2-to-CO conversion over g-C_3N_4 synthesized at different calcination temperatures under 12 h irradiation. Each condition was tested in triplicate using independently synthesized batches, and the average yields with standard deviations are summarized in Table S2.

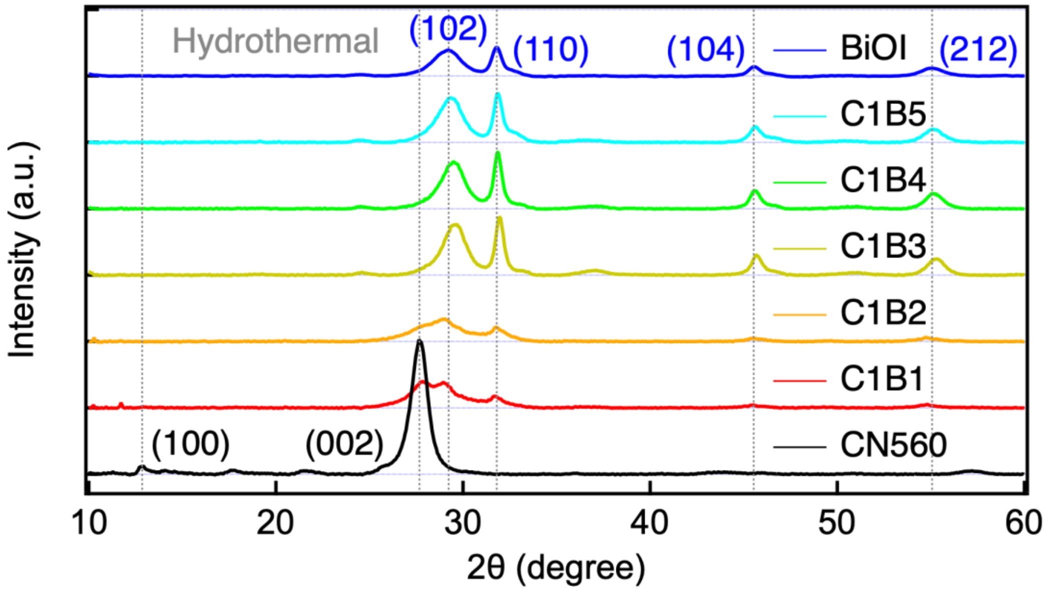

Figure S5. XRD patterns of pristine g-C_3N_4, BiOI, and their hydrothermal composites at different ratios. BiOI crystallizes in the tetragonal phase with characteristic reflections indexed to the (102), (110), (104), and (212) planes. No additional diffraction peaks or phase transformations are observed upon composite formation. The g-C_3N_4 (002) reflection remains visible, whereas the (100) reflection becomes weak or absent after composite formation, indicating disruption of in-plane ordering induced by interfacial coupling.

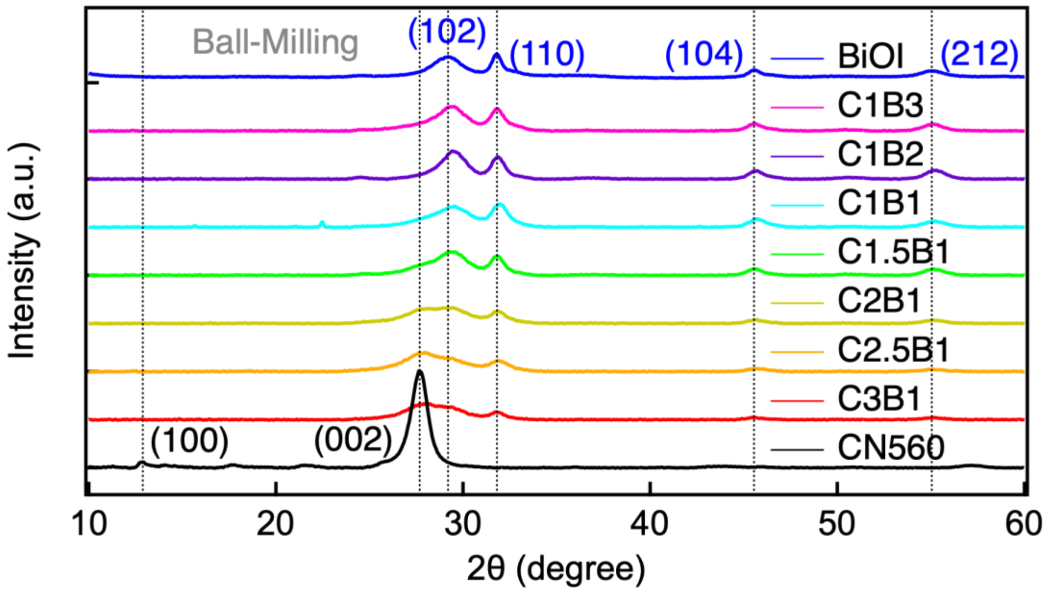

Figure S6. XRD patterns of pristine g-C_3N_4, BiOI, and their ball-milled composites at different ratios. BiOI crystallizes in the tetragonal phase with characteristic reflections indexed to the (102), (110), (104), and (212) planes. No additional diffraction peaks or phase transformations are observed upon composite formation. The g-C_3N_4 (002) reflection remains visible, whereas the (100) reflection becomes weak or absent after composite formation, indicating disruption of in-plane ordering induced by interfacial coupling.

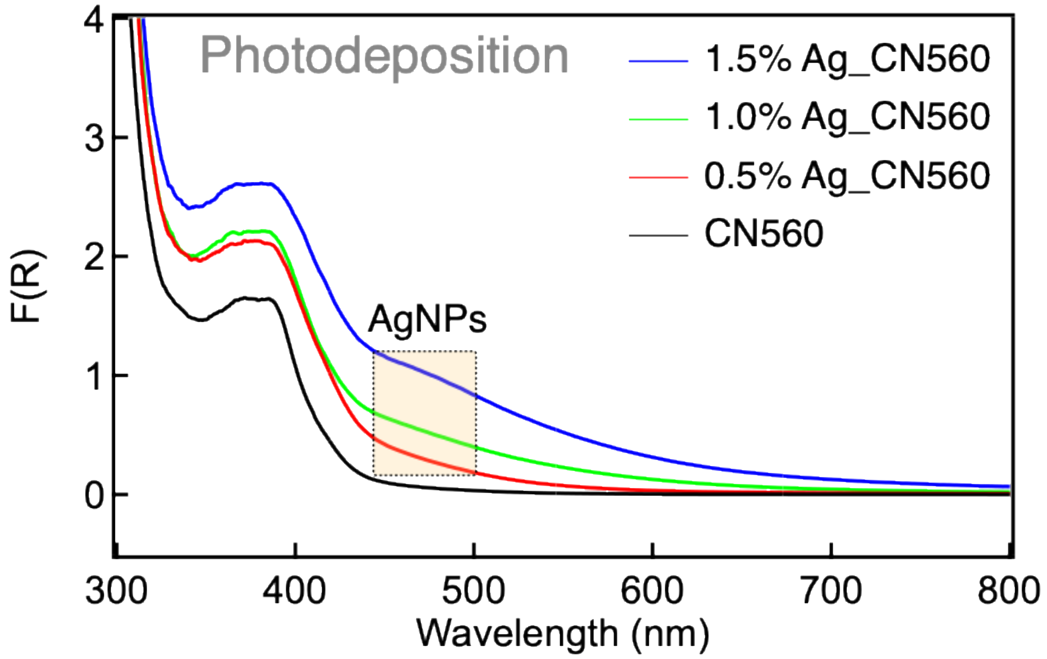

Figure S7. UV–vis diffuse reflectance spectra (DRS) of g-C_3N_4 with different Ag nanoparticles (AgNPs) photodeposition weight ratios.

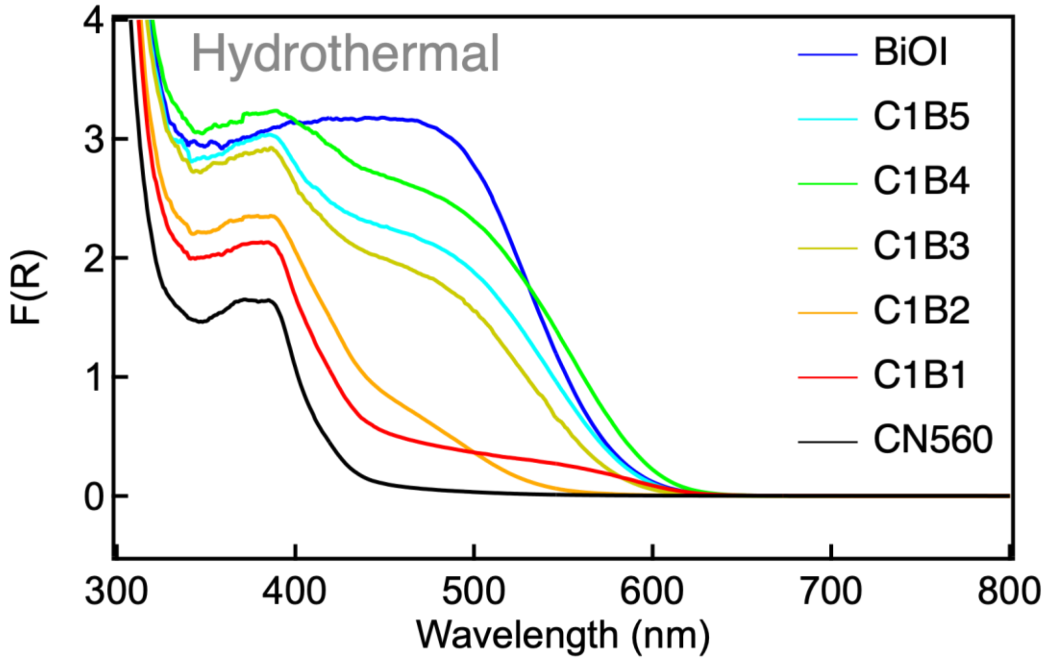

Figure S8. UV–vis diffuse reflectance spectra (DRS) of pristine g-C_3N_4 (CN560), BiOI, and their hydrothermally synthesized composites at different ratios.

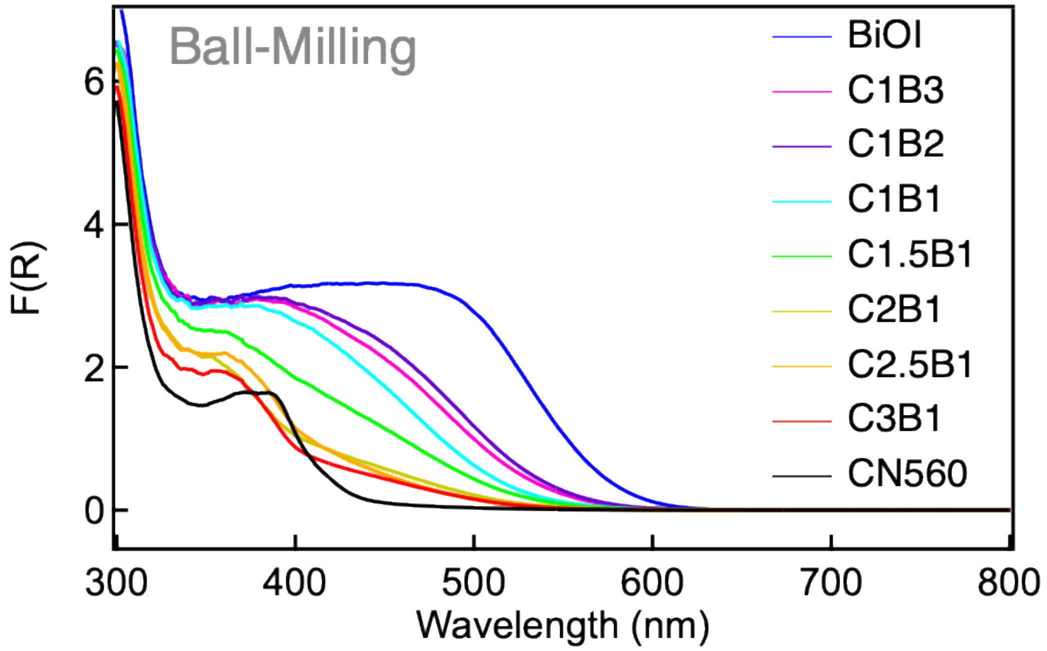

Figure S9. UV–vis diffuse reflectance spectra (DRS) of pristine g-C_3N_4 (CN560), BiOI, and their ball-milled composites at different mixing ratios.

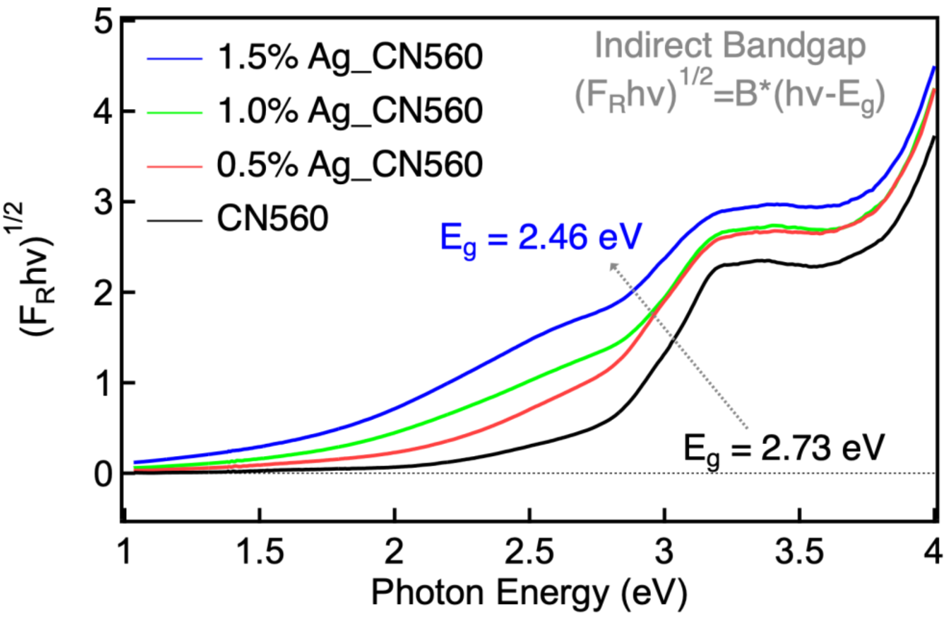

Figure S10. Tauc plots for estimating the optical bandgaps of Ag-photodeposited g-C_3N_4 samples with different Ag loading ratios, assuming indirect electronic transitions. The derived bandgap values are summarized in Table S3.

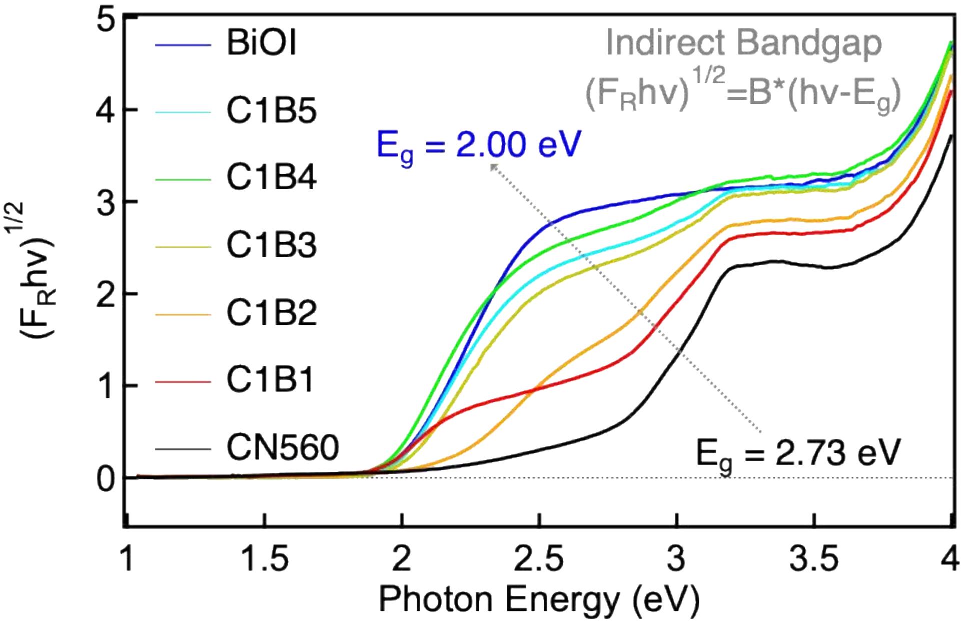

Figure S11. Tauc plots used to estimate the indirect bandgaps of pristine CN560, BiOI, and their hydrothermally synthesized composites. The derived bandgap values are summarized in Table S4.

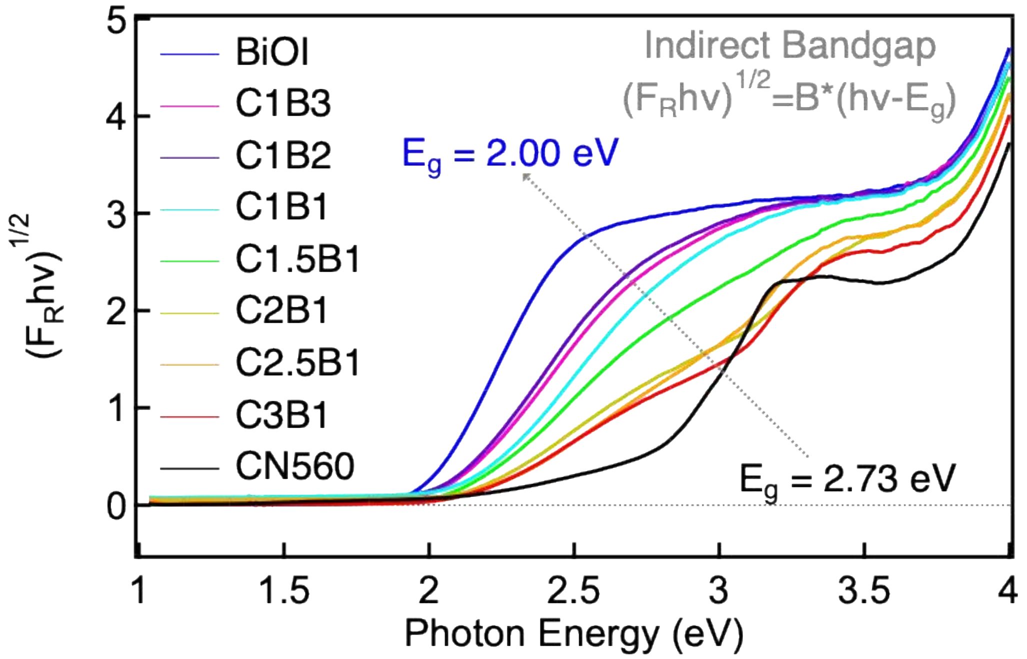

Figure S12. Tauc plots used to estimate the indirect bandgaps of pristine CN560, BiOI, and their ball-milled composites. The derived bandgap values are summarized in Table S5.

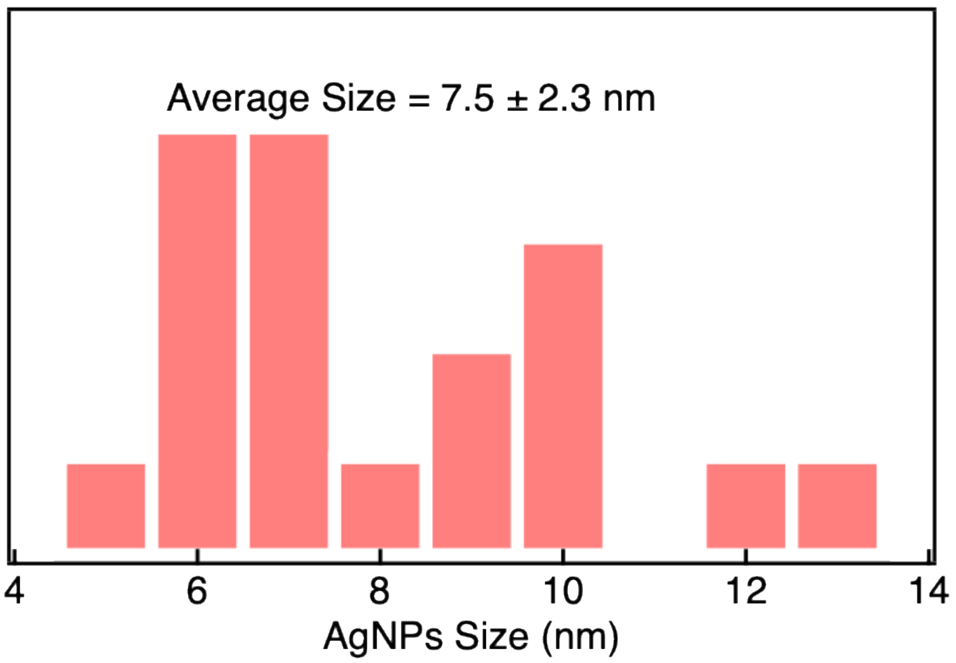

Figure S13. Particle size distribution histogram of Ag nanoparticles photodeposited on CN560, showing an average size of 7.5 ± 2.3 nm.

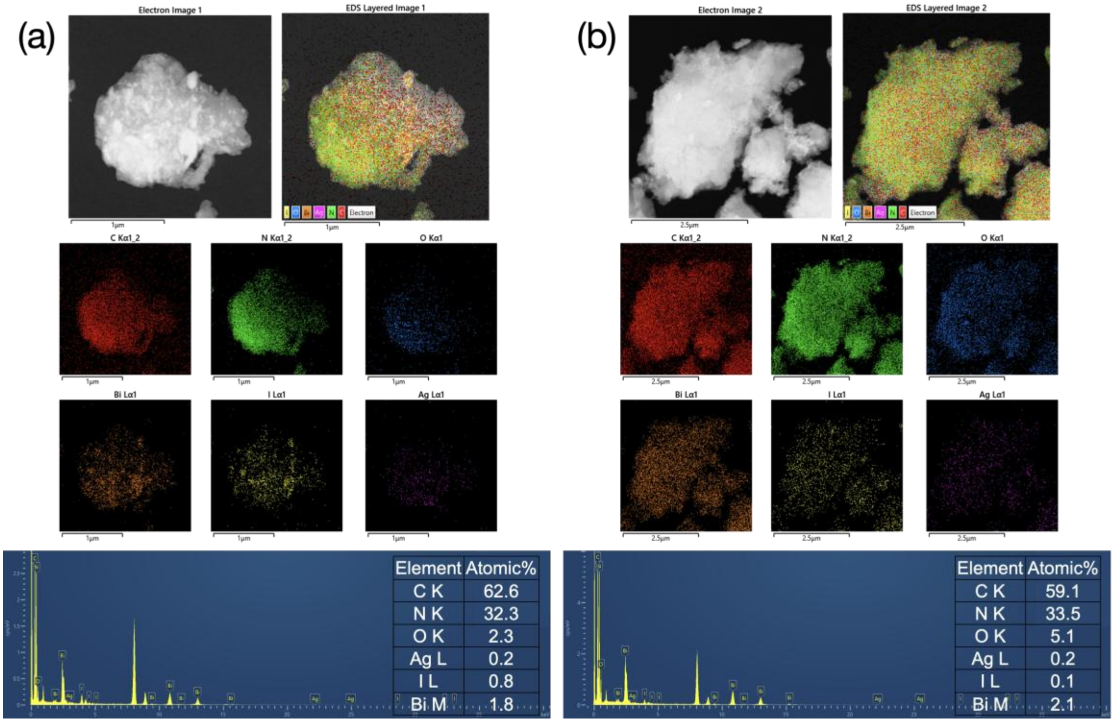

Figure S14. TEM–EDS elemental mapping of indirect Z-scheme composite (0.5AgC2.5B1), confirming the homogeneous nanoscale co-distribution of C, N, Bi, O, I, and Ag across the composite, consistent with intimate heterojunction formation.

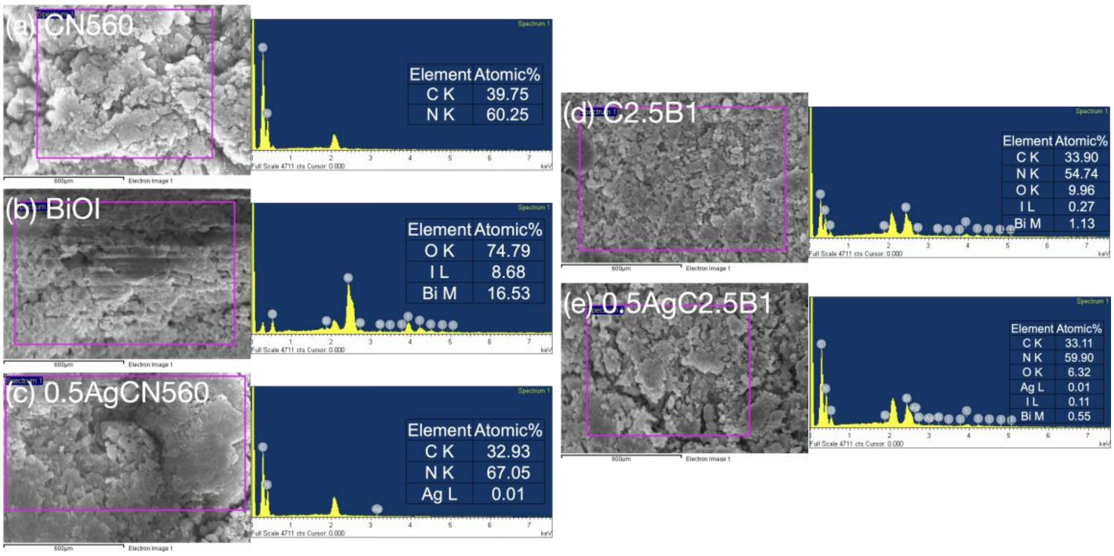

Figure S15. SEM–EDS of pristine CN560, BiOI, Ag-modified CN560 (0.5AgCN560), direct Z-scheme composite (C2.5B1), and indirect Z-scheme composite (0.5AgC2.5B1), confirming the uniform spatial distribution of C, N, Bi, O, I, and Ag across the different materials.

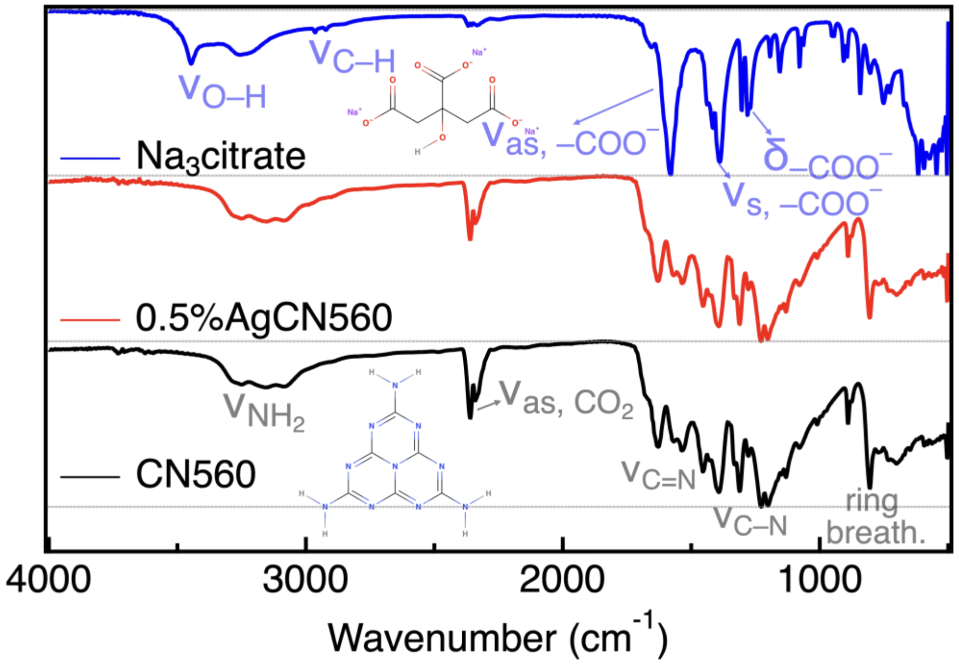

Figure S16. FT-IR spectra of pristine CN560 (black), Ag-deposited CN560 (0.5AgCN560, red), and sodium citrate (blue).

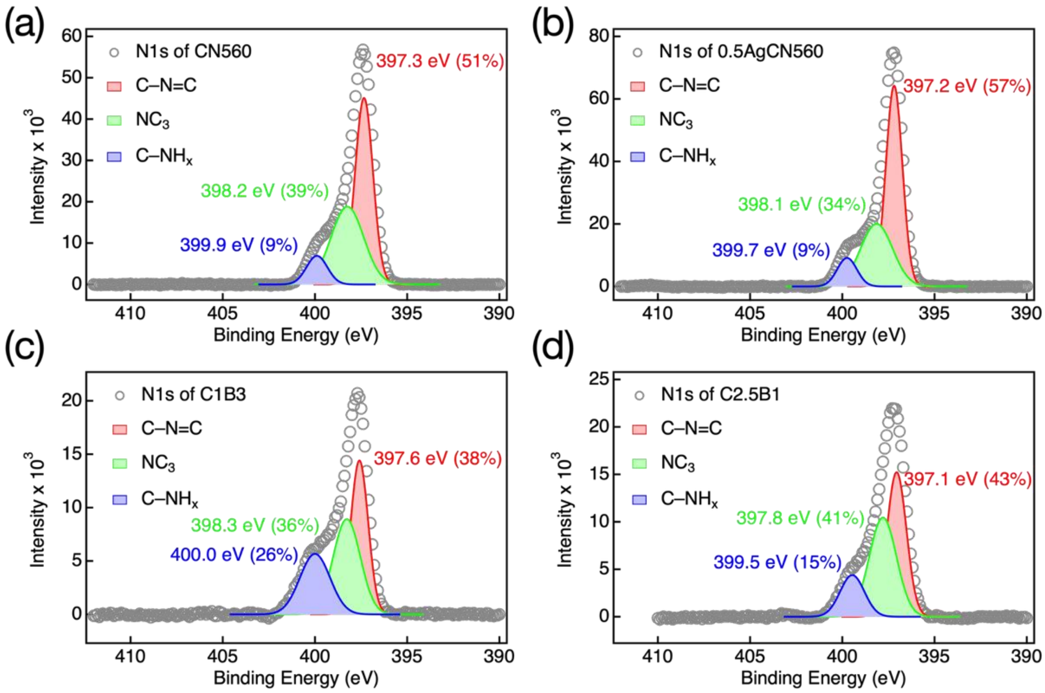

Figure S17. High-resolution N 1s XPS spectra of (a) CN560, (b) Ag-deposited CN560 (0.5AgCN560), (c) hydrothermal composite C1B3, and (d) ball-milled composite C2.5B1, deconvoluted with multiple Gaussian functions as indicated.

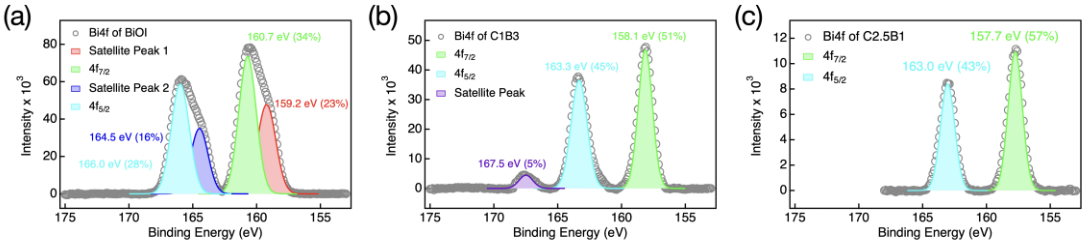

Figure S18. High-resolution Bi 4f XPS spectra of (a) pristine BiOI, (b) hydrothermal composite C1B3, and (c) ball-milled composite C2.5B1, deconvoluted using multiple Gaussian functions representing Bi $4f_{7/2}$, Bi $4f_{5/2}$, and their satellite features.

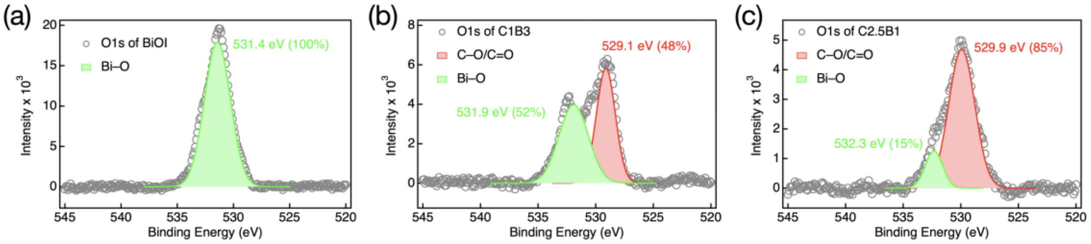

Figure S19. High-resolution O 1s XPS spectra of (a) pristine BiOI, (b) hydrothermal composite C1B3, and (c) ball-milled composite C2.5B1, deconvoluted using multiple Gaussian functions representing Bi–O and C–O/C=O contributions.

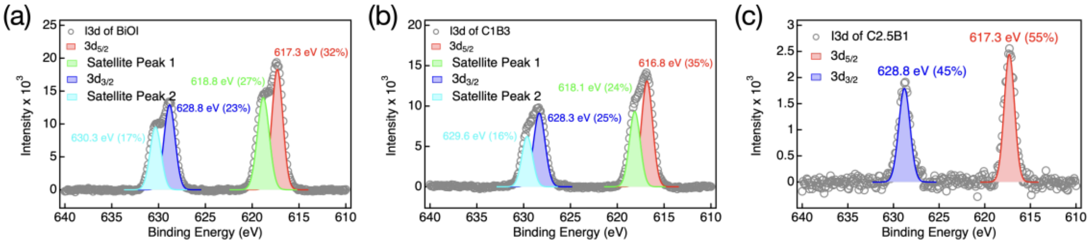

Figure S20. High-resolution I 3d XPS spectra of (a) pristine BiOI, (b) hydrothermal composite C1B3, and (c) ball-milled composite C2.5B1, fitted with multiple Gaussian components representing I $3d_{5/2}$, I $3d_{3/2}$, and satellite features.

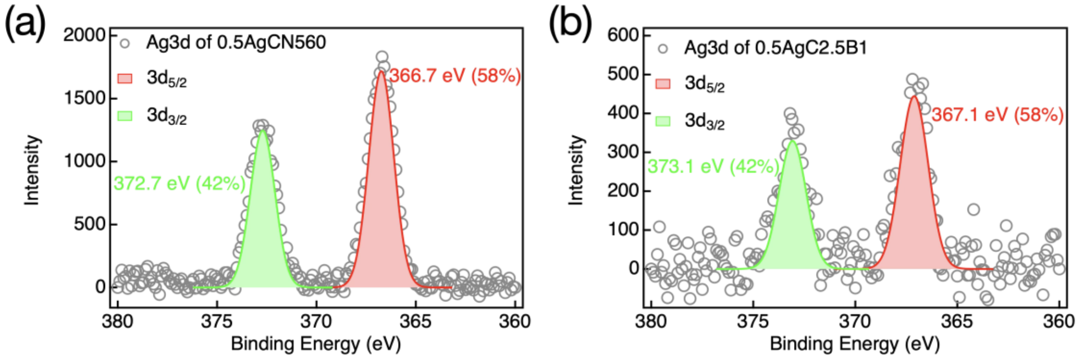

Figure S21. High-resolution Ag 3d XPS spectra of (a) Ag-deposited CN560 (0.5AgCN560) and (b) indirect Z-scheme composite (0.5AgC2.5B1), deconvoluted using two Gaussian functions representing Ag $3d_{5/2}$ and Ag $3d_{3/2}$ components.

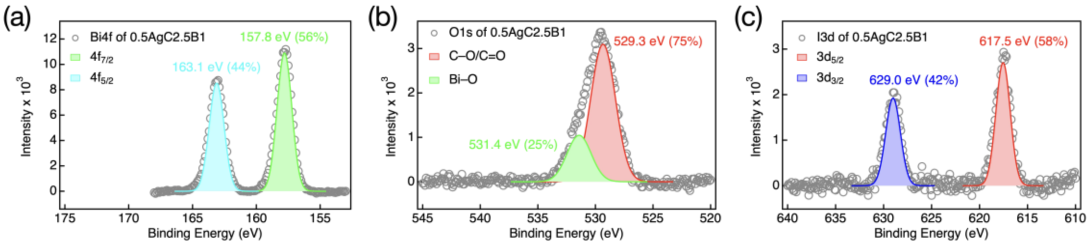

Figure S22. High-resolution XPS spectra of (a) Bi 4f, (b) O 1s, and (c) I 3d for the indirect Z-scheme composite 0.5AgC2.5B1, deconvoluted using two Gaussian functions.

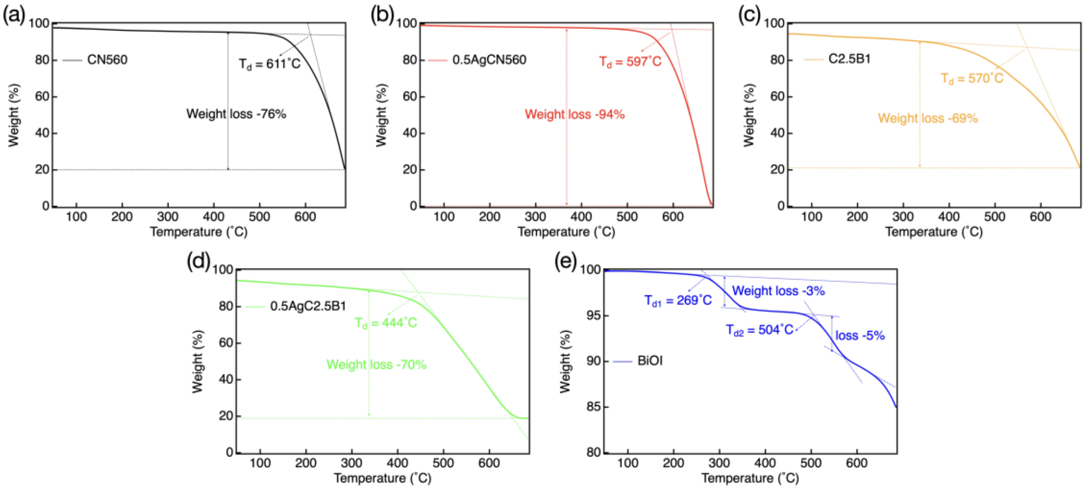

Figure S23. TGA curves of (a) CN560, (b) 0.5AgCN560, (c) C2.5B1, (d) 0.5AgC2.5B1, and (e) BiOI, showing the respective degradation onset temperatures (T_d) and total weight-loss percentages.

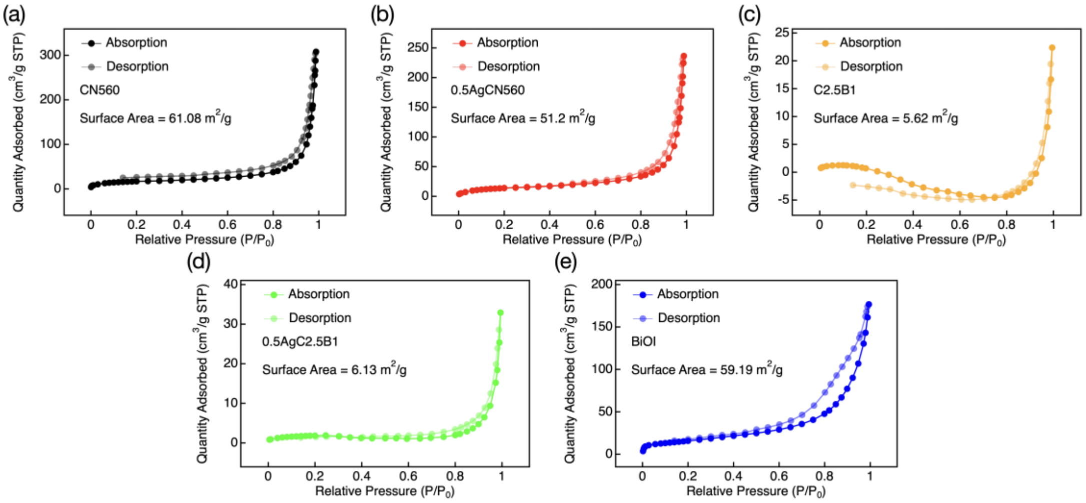

Figure S24. N_2 adsorption–desorption isotherms and BET surface area analysis of (a) CN560, (b) 0.5AgCN560, (c) C2.5B1, (d) 0.5AgC2.5B1, and (e) BiOI.

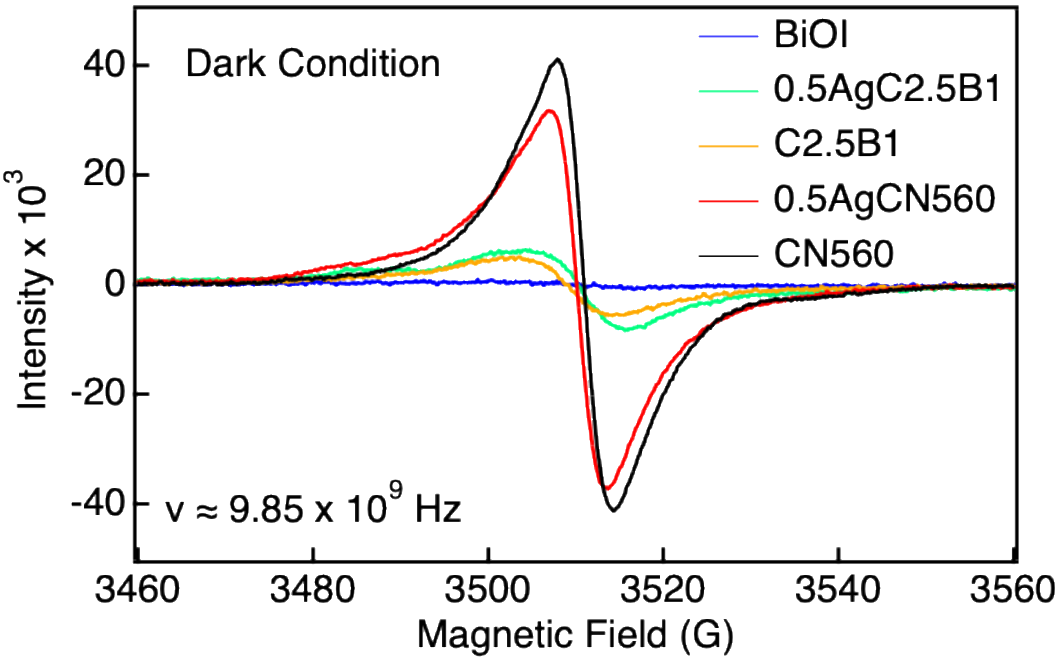

Figure S25. EPR spectra of CN560, 0.5AgCN560, C2.5B1, 0.5AgC2.5B1, and BiOI recorded under dark conditions at 9.85 GHz.

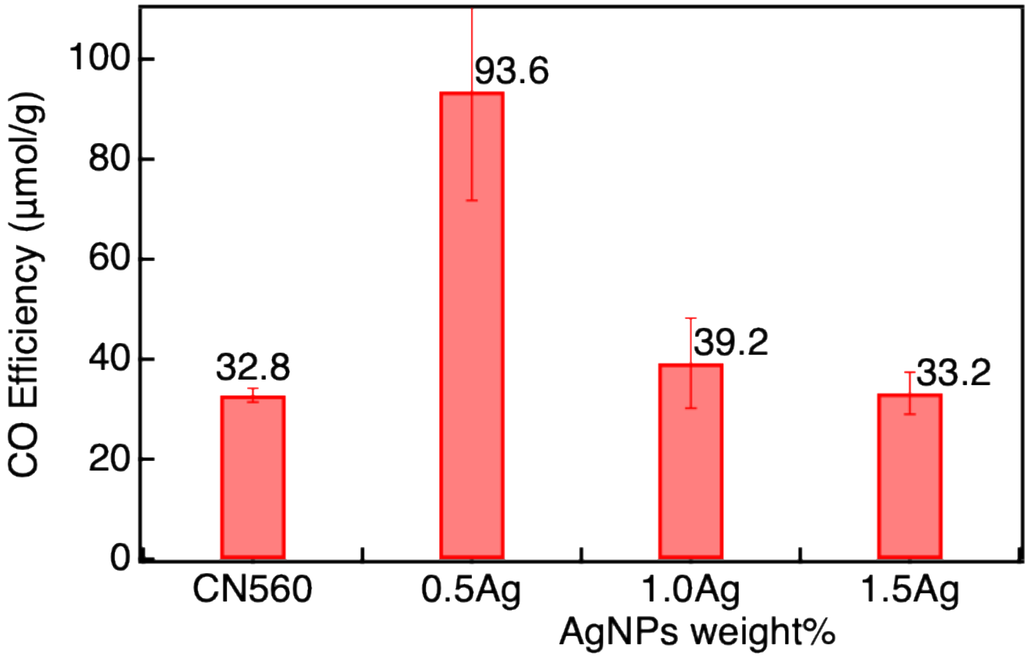

Figure S26. Photocatalytic CO_2-to-CO conversion over g-C_3N_4 loaded with different Ag nanoparticles (AgNPs) contents under 12 h illumination. Each condition was tested three times using independently prepared batches and the average yields with standard deviations summarized in Table S7.

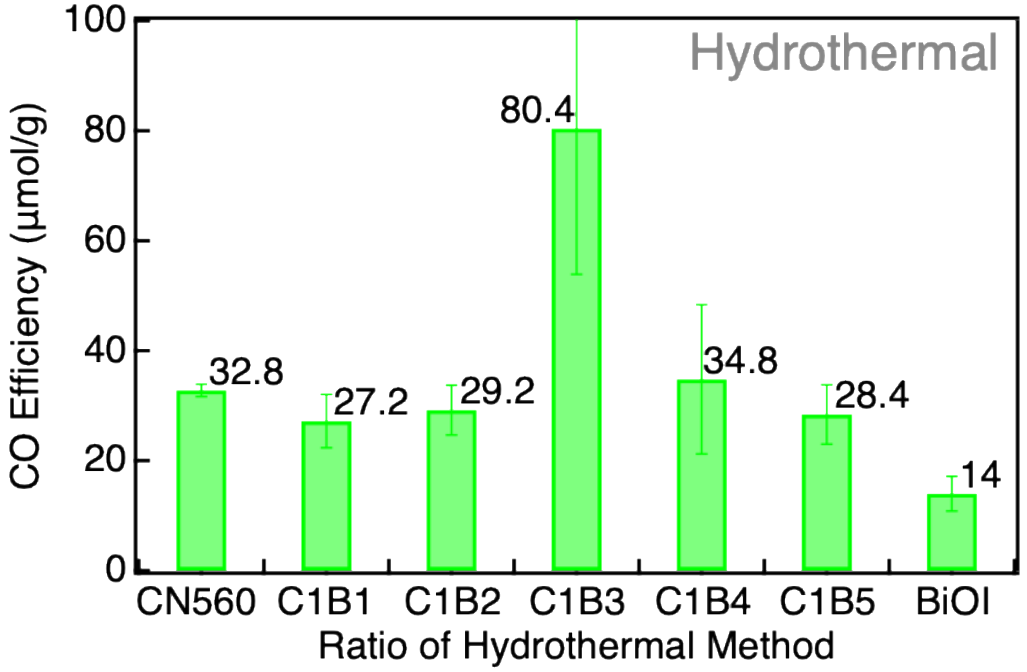

Figure S27. Photocatalytic CO_2-to-CO conversion over g-C_3N_4/BiOI composites synthesized by the hydrothermal method at various weight ratios, measured under 12 h illumination. Each condition was tested three times using independently prepared batches and average yields with standard deviations summarized in Table S8.

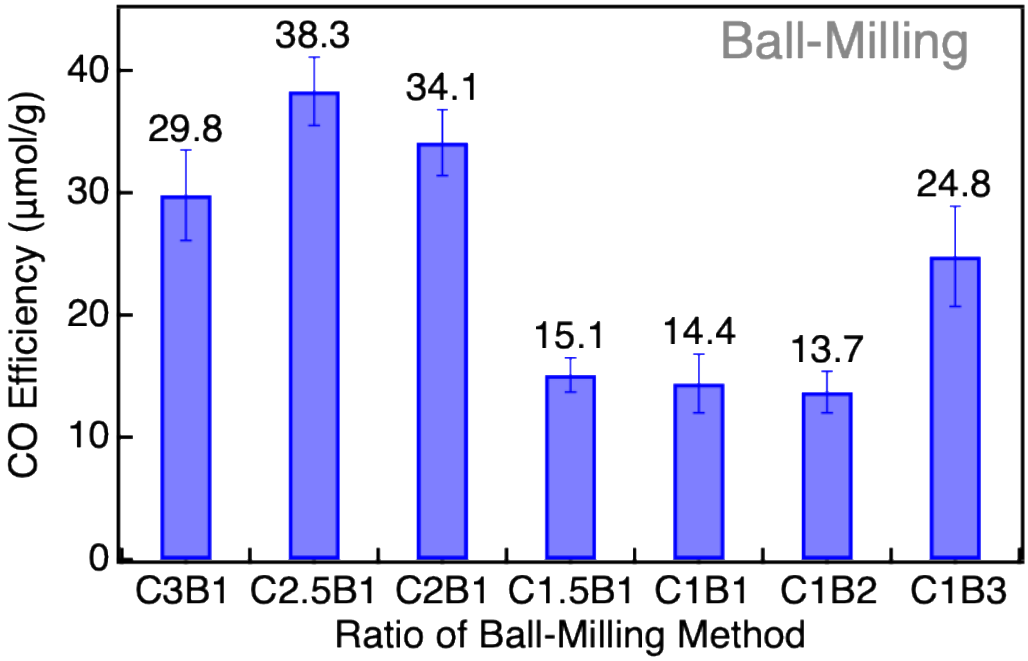

Figure S28. Photocatalytic CO_2-to-CO conversion over g-C_3N_4/BiOI composites prepared via solvent-free ball milling method at different weight ratios, measured under 6 h illumination. Each condition was tested three times using independently prepared batches and average yields with standard deviations summarized in Table S9.

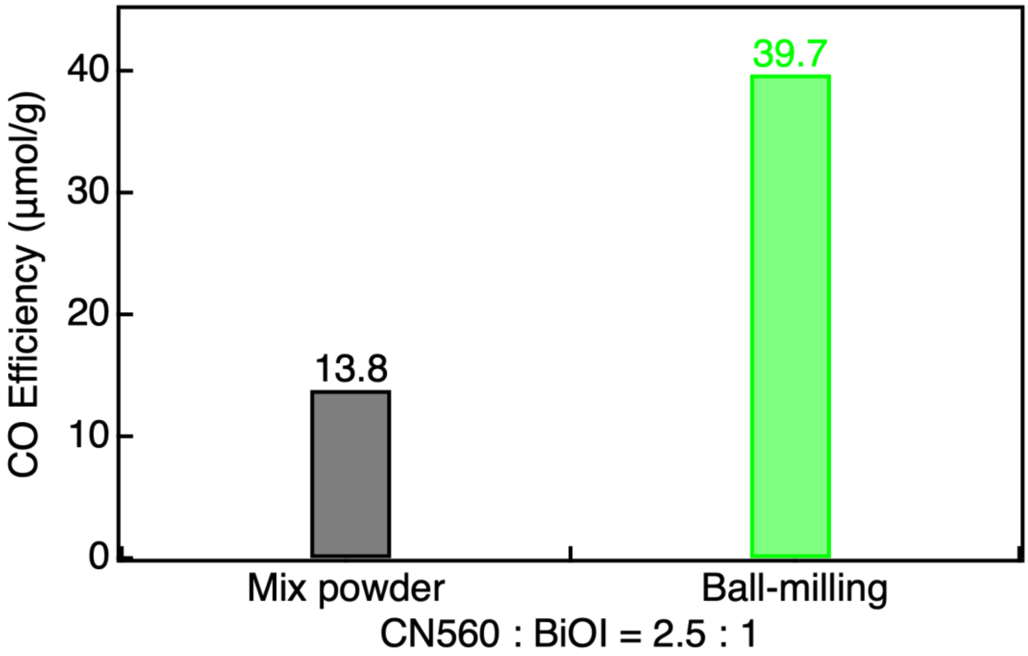

Figure S29. Photocatalytic CO_2-to-CO conversion over g-C_3N_4/BiOI composites (weight ratio = 2.5:1) prepared by simple physical mixing (black bar) and by solvent-free ball milling (green bar) under 6 h illumination.

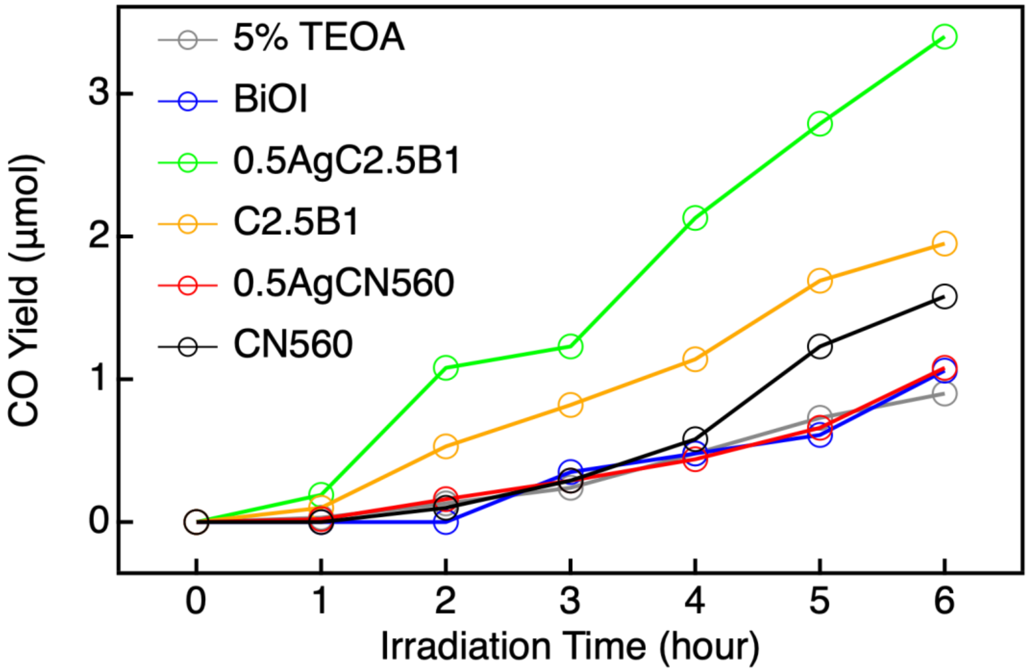

Figure S30. Time-resolved CO evolution profiles for photocatalytic CO_2 reduction under 5% TEOA without background subtraction. These uncorrected data include both the catalytic CO contribution and the CO generated from TEOA photodecomposition, which was later subtracted to obtain the net CO yields shown in Figure 5b.

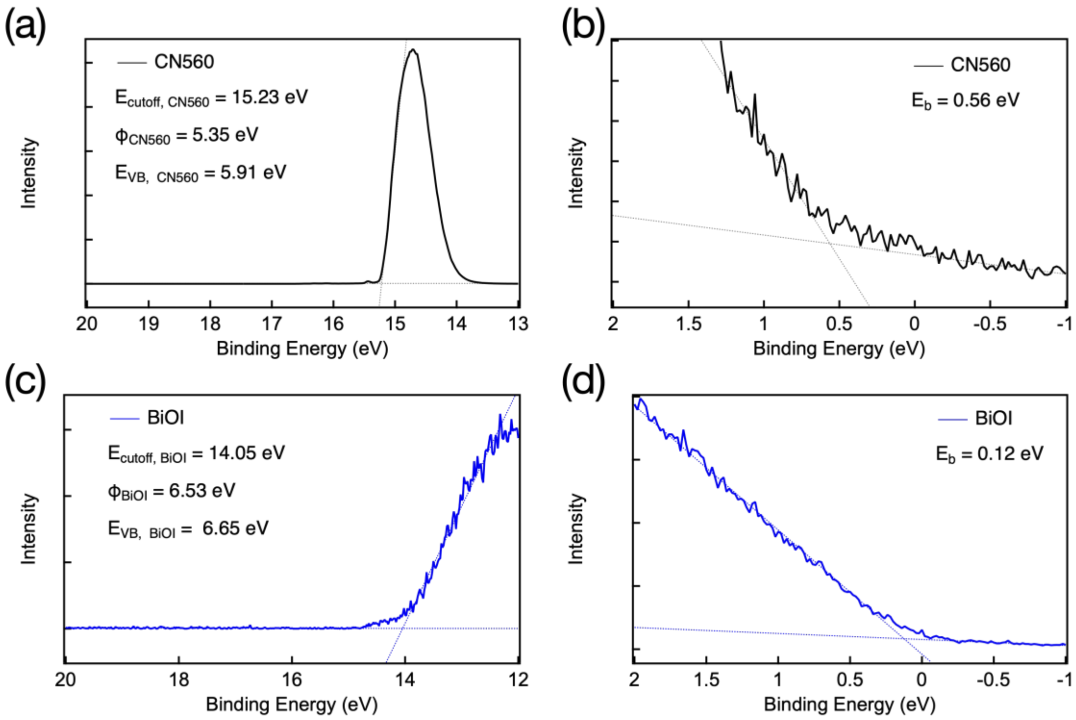

Figure S31. Ultraviolet photoelectron spectroscopy (UPS) of (a, b) CN560 and (c, d) BiOI, including work function and valance band maximum.

Table S1. Voigt function fitting parameters for g-C_3N_4 at various calcination temperatures (2θ, FWHM, d value, etc.).

Voigt Function[1]	2θ (°)	Area	G_{FWHM}	L_{FWHM}	Shape[2]	y_0	d value (Å)
**CN540**	27.5	2.3	0.4	1.3	3.5	-0.0	3.25
**CN550**	27.7	2.1	0.5	1.1	2.2	-0.0	3.22
**CN560**	27.7	1.5	0.5	0.8	1.4	-0.0	3.22
**CN570**	27.7	1.4	0.5	0.7	1.4	-0.0	3.22
**CN580**	27.7	1.5	0.5	0.7	1.3	-0.0	3.22

[1] Voigt function, $f(x) = \frac{2Area}{G_{FWHM}}\sqrt{\frac{ln(2)}{\pi}} \cdot Voigt\left[\frac{2\sqrt{ln(2)}}{G_{FWHM}}(x - x_0),\ Shape \cdot 2\sqrt{ln(2)}\right]$

[2] $Shape = \frac{L_{FWHM}}{G_{FWHM}}$

Table S2. Average CO generation rates and standard deviations for g-C_3N_4 samples synthesized at different calcination temperatures during photocatalytic CO_2 reduction. Each value represents the mean of three independent experiments conducted under identical illumination and reaction conditions.

Temp. (°C)	CO Avg (μmol·g^{-1})	CO Std (μmol·g^{-1})
**540**	69.6	31
**550**	52.4	15
**560**	32.8	1.1
**570**	32.4	5.2
**580**	46.8	6.8

Table S3. Optical bandgaps of Ag nanoparticle–photodeposited g-C_3N_4 samples, estimated from Tauc plot analysis.

%AgCN560	Bandgap (eV)
**0**	2.73
**0.5**	2.58
**1.0**	2.60
**1.5**	2.46

Table S4. Optical bandgaps of pristine g-C_3N_4, BiOI, and their hydrothermally synthesized composites, estimated from Tauc plot analysis.

Hydrothermal	Bandgap (eV)
**CN560**	2.73
**C1B1**	2.50
**C1B2**	2.27
**C1B3**	2.00
**C1B4**	1.94
**C1B5**	1.97
**BiOI**	2.00

Table S5. Optical bandgaps of ball-milled hybrid composites, estimated from Tauc plot analysis.

Ball-Milling	Bandgap (eV)
**C3B1**	2.62
**C2.5B1**	2.58
**C2B1**	2.45
**C1.5B1**	2.10
**C1B1**	2.14
**C1B2**	2.07
**C1B3**	2.07

Table S6. Summary of photocatalytic CO_2-to-CO conversion performance of CN560, BiOI, 0.5AgCN560, C2.5B1 (direct Z-scheme), and 0.5AgC2.5B1 (indirect Z-scheme) under both water and triethanolamine (TEOA) environments after 6 h irradiation. CO yields were averaged from three independent experiments, and background CO formation from TEOA photolysis was subtracted for accuracy.

	**Water**	**TEOA**	
**Sample**	**CO Avg ($\mu mol \cdot g^{-1}$)**	**CO Avg ($\mu mol \cdot g^{-1}$)**	**CO Std ($\mu mol \cdot g^{-1}$)**
**CN560**	28	144.5	16
**BiOI**	11.5	68.2	2.3
**0.5AgCN560**	29.6	137.2	52
**C2.5B1**	39.7	180.8	30
**0.5AgC2.5B1**	40.3	344.6	32

Table S7. Average CO production efficiency and standard deviations for g-C_3N_4 samples photodeposited with different Ag nanoparticle loadings during CO_2 photoreduction for 12 h irradiation.

**%AgCN560**	**CO Avg ($\mu mol \cdot g^{-1}$)**	**CO Std ($\mu mol \cdot g^{-1}$)**
**0**	32.8	1.4
**0.5**	93.6	22
**1.0**	39.2	9.0
**1.5**	33.2	4.2

Table S8. Average CO production efficiency and standard deviations for g-C_3N_4/BiOI composites prepared by hydrothermal synthesis at various mixing ratios during CO_2 photoreduction for 12 h irradiation.

Hydrothermal	CO Avg ($\mu mol \cdot g^{-1}$)	CO Std ($\mu mol \cdot g^{-1}$)
**CN560**	32.8	1.1
**C1B1**	27.2	4.8
**C1B2**	29.2	4.5
**C1B3**	80.4	27
**C1B4**	34.8	14
**C1B5**	28.4	5.4
**BiOI**	14	3.1

Table S9. Average CO production efficiency and standard deviations for g-C_3N_4/BiOI composites fabricated via solvent-free ball-milling method at different weight ratios during CO_2 photoreduction for 6 h irradiation.

Ball-Milling	CO Avg (μmol·g^{-1})	CO Std (μmol·g^{-1})
**C3B1**	29.8	3.7
**C2.5B1**	38.3	2.8
**C2B1**	34.1	2.7
**C1.5B1**	15.1	1.4
**C1B1**	14.4	2.4
**C1B2**	13.7	1.7
**C1B3**	24.8	4.1

Table S10. Comparison of representative g-C3N4-based direct, indirect, and S-scheme photocatalysts for liquid-phase and gas-phase photocatalytic CO2 reduction under reported conditions, including the Ag-bridged indirect Z-scheme developed in this work.

Photocatalyst	Condition	Light source	Product / yield	Ref.
Indirect<br>BiOI/ g-C_3N_4	CO_2 and H_2O	300 W xenon arc<br>lamp (λ > 400 nm)	CO 17.23 μmol/g<br>CH_4 0.82 μmol/g	[4]
S-scheme<br>BiOI/g-C_3N_4	50 mL TEOA (10%<br>v/v) + CO_2	300 W xenon lamp<br>(λ > 400 nm)	CO<br>12.45 μmol/g	[5]
Direct Z-Scheme<br>Ag_3PO_4/g-C_3N_4	4 mL H_2O + CO_2	500 W xenon lamp<br>(λ > 420 nm)	CO<br>57.5 μmol/g/h	[6]
Direct Z-Scheme<br>g-C_3N_4/$FeWO_4$	20 mL Na_2SO_3<br>(0.5 M)	solar simulator	CO<br>30.6 μmol/g	[7]
Direct Z-scheme<br>g-C_3N_4/ZnO	1 mmol $NaHCO_3$<br>H_2SO_4 (2M, 0.3 mL)	350 W xenon lamp	CH_3OH<br>1.32 μmol/g/h	[8]
S-scheme<br>BiOI/CF/g-C_3N_4	1 mmol $NaHCO_3$<br>H_2SO_4 (2M, 0.3 mL)	300 W xenon lamp<br>(λ > 400 nm)	CO<br>458 μmol/h/m^2	[9]
Ternary indirect Z-scheme<br>g-C_3N_4/AgNPs/BiOI	2 mL TEOA (5%<br>v/v) + CO_2 + H_2O	solar simulator	CO<br>344.6 μmol/g	**This work**

Reference

[1] G. Zhang, J. Zhang, M. Zhang, X. Wang, *J. Mater. Chem.* **2012**, *22*, 8083-8091.

[2] K. Wenderich, G. Mul, *Chem. Rev.* **2016**, *116*, 14587-14619.

[3] J. Xia, S. Yin, H. Li, H. Xu, Y. Yan, Q. Zhang, *Langmuir* **2011**, *27*, 1200-1206.

[4] J.-C. Wang, H.-C. Yao, Z.-Y. Fan, L. Zhang, J.-S. Wang, S.-Q. Zang, Z.-J. Li, *ACS Appl. Mater. Interfaces* **2016**, *8*, 3765-3775.

[5] H. Li, D. Wang, C. Miao, F. xia, Y. Wang, Y. Wang, C. Liu, G. Che, *J. Environ. Chem. Eng.* **2022**, *10*, 108201.

[6] Y. He, L. Zhang, B. Teng, M. Fan, *Environ. Sci. amp; Technol.* **2015**, *49*, 649-656.

[7] R. Bhosale, S. Jain, C. P. Vinod, S. Kumar, S. Ogale, *ACS Appl. Mater. Interfaces* **2019**, *11*, 6174-6183.

[8] N. Nie, L. Zhang, J. Fu, B. Cheng, J. Yu, *Appl. Surf. Sci.* **2018**, *441*, 12-22.

[9] C. Yang, Q. Zhang, W. Wang, B. Cheng, J. Yu, S. Cao, *Sci. China Mater.* **2024**, *67*, 1830-1838.
